# Biodiversity enhances ecosystem multifunctionality across trophic levels and habitats

**DOI:** 10.1038/ncomms7936

**Published:** 2015-04-24

**Authors:** Jonathan S. Lefcheck, Jarrett E. K. Byrnes, Forest Isbell, Lars Gamfeldt, John N. Griffin, Nico Eisenhauer, Marc J. S. Hensel, Andy Hector, Bradley J. Cardinale, J. Emmett Duffy

**Affiliations:** 1 Department of Biological Sciences, Virginia Institute of Marine Science, College of William and Mary, Gloucester Point, Virginia 23062-1346, USA; 2 Department of Biology, University of Massachusetts Boston, Boston, Massachusetts 02125, USA; 3 Department of Ecology, Evolution and Behavior, University of Minnesota, Saint Paul, Minnesota 55108, USA; 4 Department of Biological and Environmental Sciences, University of Gothenburg, Box 461, SE-40530 Gothenburg, Sweden; 5 Department of Biosciences, Wallace Building, Swansea University, Singleton Park, Swansea SA2 8PP, UK; 6 German Centre for Integrative Biodiversity Research (iDiv) Halle-Jena-Leipzig, Deutscher Platz 5e, 04103 Leipzig, Germany; 7 Institute of Biology, University of Leipzig, Johannisallee 21, 04103 Leipzig, Germany; 8 Department of Plant Sciences, University of Oxford, OX1 3RB Oxford, UK; 9 School for Natural Resources and Environment, University of Michigan, Ann Arbor, Michigan 48109, USA; 10 Tennenbaum Marine Observatories Network, Smithsonian Institution, Washington, Washington DC 20013-7012, USA

## Abstract

The importance of biodiversity for the integrated functioning of ecosystems remains unclear because most evidence comes from analyses of biodiversity's effect on individual functions. Here we show that the effects of biodiversity on ecosystem function become more important as more functions are considered. We present the first systematic investigation of biodiversity's effect on ecosystem multifunctionality across multiple taxa, trophic levels and habitats using a comprehensive database of 94 manipulations of species richness. We show that species-rich communities maintained multiple functions at higher levels than depauperate ones. These effects were stronger for herbivore biodiversity than for plant biodiversity, and were remarkably consistent across aquatic and terrestrial habitats. Despite observed tradeoffs, the overall effect of biodiversity on multifunctionality grew stronger as more functions were considered. These results indicate that prior research has underestimated the importance of biodiversity for ecosystem functioning by focusing on individual functions and taxonomic groups.

## Abstract (web summary)

The influence of biodiversity on multiple ecosystem processes is not well understood. Analysing 94 biodiversity-ecosystem functioning experiments, Lefcheck *et al*. find that increased species richness maintains more ecological functions, across multiple taxa, trophic levels and habitats.

## Graphical abstract

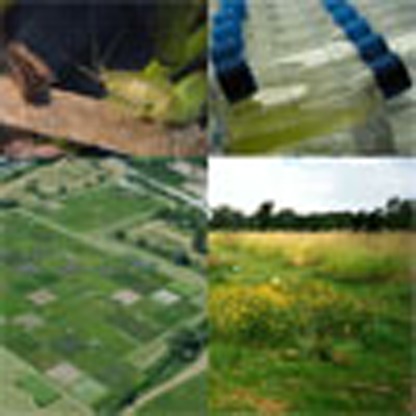

Many recent syntheses have highlighted the importance of biodiversity for maintaining stable and productive ecosystems^1^,^2^,^3^,^4^,^5^,^6^. Yet evidence for this conclusion comes mostly from experiments testing the effects of species richness on individual ecosystem functions^7^,^8^, despite the fact that natural ecosystems are defined by many interconnected processes. Moreover, these syntheses typically focus on a few ecosystem functions related to biomass production, resource use and decomposition^2^,^5^,^6^, which has led some to question the overarching conclusion that biodiversity enhances the functioning of whole ecosystems^9^,^10^. Natural systems perform many functions, all of which have the potential to be positively or negatively affected by biodiversity, or to enhance or inhibit the provision of other functions^11^. Extrapolating positive results from a single function to infer the role of biodiversity in complex systems ignores the interplay among functions^12^,^13^, as well as our desire to simultaneously extract multiple goods and services from high-functioning ecosystems^14^. Solving these problems requires considering how biodiversity simultaneously affects the multitude of ecosystem functions present in nature, which we define as *ecosystem multifunctionality*.

The complex interactions among species and functions raise two opposing possibilities when investigating the relationship between species richness (hereafter, ‘biodiversity') and ecosystem multifunctionality. First, species may appear more functionally unique as more processes are considered, leading to a stronger effect of biodiversity across multiple functions^15^,^16^,^17^,^18^. Alternatively, tradeoffs among functions may decrease multifunctionality if some functions must be low for others to be high^11^,^13^,^19^. For instance, it has been shown that the biomass of wood and non-timber forest products cannot be simultaneously maximized in Swedish forests^19^. Syntheses of biodiversity-function studies have yet to incorporate such tradeoffs. Finally, the few studies that have investigated the relationship between biodiversity and ecosystem multifunctionality have been conducted almost entirely in communities of temperate grassland plants^13^,^18^,^20^,^21^,^22^,^23^, with a few on bacterial biofilms^24^,^25^. If we wish to understand the general consequences of biodiversity loss for ecosystem multifunctionality, then it is imperative to extend this framework to other organisms, habitats and a wider range of functions^26^.

We conducted the first comprehensive test for a general relationship between biodiversity and ecosystem multifunctionality by assembling data from 94 published experiments from a broad range of taxa and ecosystems. Each experiment manipulated the richness of ≥3 species in one of five groups: dead organic matter, detritivores, primary producers, herbivores and carnivores, with studies evenly divided between aquatic and terrestrial habitats. The authors then measured the effect of biodiversity on between 2 and 12 ecosystem functions. We quantified the integrated effect of biodiversity using two complementary approaches (Fig. 1). First, we calculated how biodiversity affected the number of functions performing at high, medium and low levels, based on whether they exceeded a threshold (Fig. 1b,c)^12^,^13^,^23^,^27^. We set the threshold as a percentage of the highest observed mean level of functioning across all treatments in an experiment. This approach has been criticized because the choice of any single threshold is arbitrary, so we calculated the biodiversity effect along the entire continuum of possible thresholds from 1 to 99%^23^,^28^. We also quantified the effect of biodiversity on the average standardized yield across all functions (Fig. 1d)^20^,^21^,^22^,^23^,^29^,^30^. In both approaches, we used generalized linear mixed effects models (GLMMs) to quantify the relationship between species richness and multifunctionality. We find that biodiversity generally increased ecosystem multifunctionality, whether quantified as the number of functions performing at a threshold or as the average standardized yield, and that this effect was generally strongest for herbivores.

## Results and Discussion

### Threshold approach

**Figure 2:**
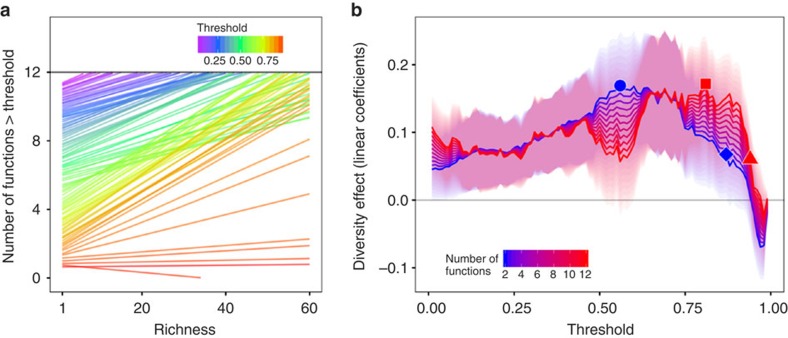
A threshold-based approach to multifunctionality revealed strong effects of biodiversity, which increased with an increasing number of functions. (**a**) The raw number of functions greater than a threshold against richness, for thresholds ranging from 1% of the maximum (purple lines) to 99% of the maximum (red lines). Lines are predicted fits from GLMMs that fixed the covariate number of functions at 12. (**b**) The biodiversity effect (expected change in the number of functions exceeding a threshold with addition of one species) against threshold. Each point corresponds to the slope of a single regression of the number of functions greater than a given threshold against richness, from 2 (blue) to 12 (red) numbers of functions. The red line corresponding to 12 functions summarizes the slopes of each line from **a**. Shaded regions represent 95% confidence intervals. The circle represents the threshold of the maximum biodiversity effect observed for two functions (56%), and the square the threshold of maximum biodiversity effect for 12 functions (81%). The diamond represents the threshold at which the biodiversity effect was still significantly positive for two functions (87%), and the triangle the threshold for 12 functions (94%).

Increasing species richness generally raised the number of functions performing above a threshold, regardless of how many functions were measured (Fig. 2 and Supplementary Fig. 1). In the case of 12 functions—the maximum number from any study and arguably the most realistic approximation of a multifunctional ecosystem in our data set—single species were unable to sustain all functions at even a 1% threshold, indicated by the intercepts of the fitted lines from a GLMM failing to reach the value of 12 (Fig. 2a). This trend suggests that there were tradeoffs among many functions in species monocultures. The same model predicted that the most diverse mixtures could sustain all 12 functions at 81% of their maximum values, as indicated by the fitted lines crossing 12 functions at this threshold (Fig. 2a). This finding suggests that different functions are often maximized by different species and that, consequently, only diverse mixtures provide the species or combinations necessary to maximize multiple functions^13^. The inability of diverse treatments to sustain all functions at their maximum values (beyond 81% of their maximum for the 12 function example above) further implies there are tradeoffs among functions as well, such that even diverse communities cannot simultaneously maximize every function^11^,^12^,^13^,^19^. Although only a single study in our analysis measured 12 functions^31^, the GLMM incorporates variation for all studies in the predicted fits, thus representing the predicted effect had any given experiment measured 12 functions. The predictions for large numbers of functions are thus speculative, as this extrapolates past the range of most of our data. Nevertheless, the effect of biodiversity in bringing multiple functions close to their maximum is also clearly evident for experiments that measured fewer functions (Supplementary Fig. 1). For instance, the most diverse assemblages in experiments that measured only two functions (*N*=41) were predicted to sustain both functions at 79% of their maximum (Supplementary Fig. 1a), very similar to the 81% threshold identified in Fig. 2a.

**Figure 3:**
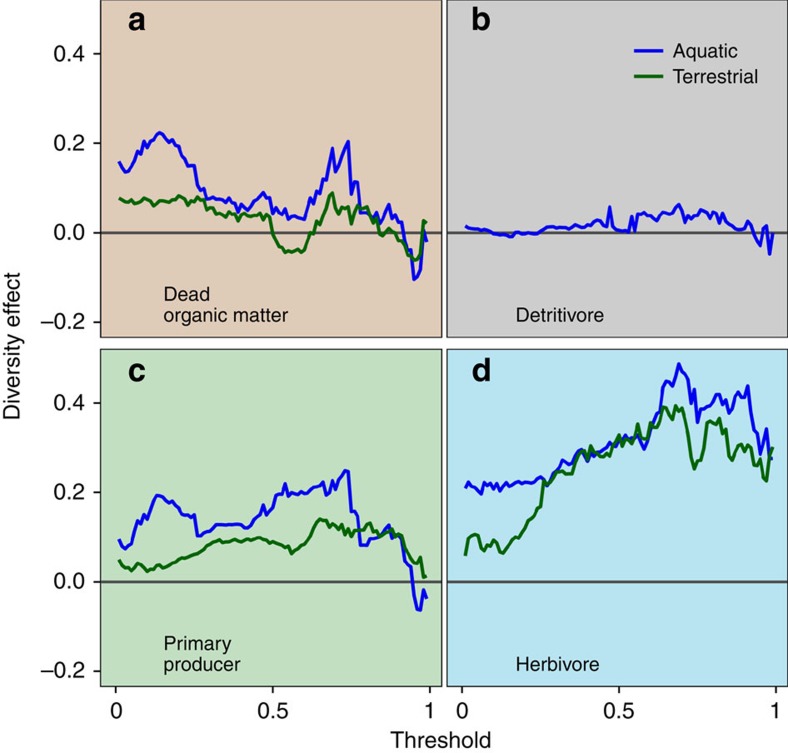
Comparison of the effect of richness on the number of functions above a threshold revealed strongest effects for herbivores, and consistent effects across habitat. The effect of biodiversity (linear coefficient regressing the number of functions above a threshold against richness) plotted against the continuum of thresholds from 1 to 99% of the maximum observed level of functioning, for (**a**) dead organic matter, (**b**) detritivores, (**c**) primary producers and (**d**) herbivores. Blue lines represent trends from experiments conducted in aquatic systems, and green lines from terrestrial systems.

Biodiversity had stronger effects as more functions were considered. Plotting the effect sizes from the mixed models (the linear coefficients representing the effect of biodiversity on multifunctionality) against threshold showed that biodiversity always had a significant positive or neutral effect but never a significant negative effect on multifunctionality, regardless of how many functions were measured (Fig. 2b). Moreover, the largest biodiversity effect sizes occurred at increasingly higher threshold as more functions were considered (Fig. 2b, Supplementary Fig. 2). For example, when only two functions were considered, the largest biodiversity effect occurred at a threshold of 56% (circle, Fig. 2b). But when considering 12 functions, the largest biodiversity effect occurred at the 81% threshold (square, Fig. 2b). Moreover, the threshold at which biodiversity still had a significant positive effect—where the slope is significantly greater than zero—increased as more functions were considered (Fig. 2b and Supplementary Fig. 3). For two functions, biodiversity ceased having an effect after the 87% threshold (diamond, Fig. 2b), but for 12 functions, biodiversity continued to increase the number of functions greater than a threshold up until 94% of the maximum (triangle, Fig. 3b).

### Differences by trophic level

A striking feature of our analysis was the stronger effects of biodiversity on multifunctionality among herbivores than among plants (Fig. 3). Experiments that manipulated detritivores (*N*=16) and dead organic matter (*N*=12) showed the weakest effects of biodiversity (Fig. 3a,b). Experiments that manipulated plant biodiversity (*N*=47) had stronger effects, but they tapered off at high thresholds (Fig. 3c). Only experiments that manipulated herbivores (*N*=16) revealed a strong positive effect of biodiversity even at the highest threshold (Fig. 3d). Carnivores exhibited more variability (Supplementary Fig. 5), but we have little confidence in this pattern due to the extremely small sample size (*N*=3). These differences among trophic groups were consistent across both aquatic and terrestrial habitats, implying a high degree of generality. This result may be an artefact of higher overall species richness in manipulations of plants than of herbivores in our data set. However, even when we remove all treatments that exceed the maximum level of richness manipulated for herbivores (*S*≤6), the trend still holds (Supplementary Fig. 6). This finding supports conceptual predictions that biodiversity loss should have greater consequences for higher trophic levels, in part because animals typically utilize more diverse resource pools and have more complex behaviours than do plants^33^,^34^. This result also agrees with findings from recent meta-analyses comparing consumer biodiversity effect sizes with those for primary producers^35^,^36^. Finally, it suggests that previous investigations of multifunctionality focused on temperate grassland plants may underestimate the importance of biodiversity for multifunctionality across whole food webs^15^,^18^,^21^,^22^,^23^.

### Functional turnover

One possible explanation for the increasing importance of biodiversity as more functions are measured might be that functions are correlated, and reflect the same underlying process that responds positively to biodiversity. This cannot explain our results, however, as the mean pairwise rank correlations among functions in our data set was *r*=0.19, and tended to decline with an increasing number of functions (Supplementary Fig. 4). An alternative explanation is that species are complementary with respect to their effect on functioning, and this complementarity only manifests where there are more opportunities (that is, more functions) for the biological differences among species to be expressed^15^,^18^. To test this hypothesis, we derived an index quantifying the proportion of functions maximized or minimized by different species in monoculture^18^. Across all studies, 69% (±0.02) of functions were maximized and 71% (±0.02) were minimized by different species. This suggests that no one species consistently promoted (or decreased) all functions, but instead unique sets of species were necessary to maximize (or minimize) multifunctionality. Although performance may well differ in mixed-species assemblages^32^, which we cannot test with our summarized data, similar complementarity has been observed in diverse assemblages where species can interact^15^,^18^,^23^.

### Simulation study

Examination of effect sizes from each study revealed that, in a number of individual cases, biodiversity had a negative effect on multifunctionality (Supplementary Fig. 7). On average, however, the proportion of significantly positive effects was greater than the proportion of negative effects, both of which tended to increase with more functions (Supplementary Fig. 8). The increase in negative effects as more functions were measured may reflect the increasing frequency of tradeoffs among functions as biodiversity increases in nature. Alternatively, it may also be a consequence of sampling, or a limitation of the short-time scales on which many experimental manipulations in our data set were conducted. Biodiversity manipulations that have measured many functions have noted a decrease in the proportion of negative effects through time^37^. Along a similar line, our analysis revealed a dip in the biodiversity effect around the 50% threshold, particularly when many functions were measured (Fig. 2b). We hypothesized that this dip was due to functions negatively affected by biodiversity no longer influencing the analysis after intermediate thresholds. To test this hypothesis, we conducted exploratory simulations varying the proportion of positive to negative biodiversity effects on individual functions, and repeated our prior threshold analyses. We found that increasing the proportion of negative effects caused the relationship between linear coefficients and thresholds to switch from concave-down to concave-up (Supplementary Fig. 9), with a slightly higher proportion of positive effects leading to a dip, as observed in the empirical data set. These results suggest that, while negative biodiversity–ecosystem functioning relationships are present in our data set and are important, they are not sufficient to cause the overall relationship to decline.

### Averaging approach

The use of an average standardized index across all functions has often been used to quantify multifunctionality in the past^20^,^21^,^22^,^29^, but has come under increasing criticism recently as such an approach cannot readily identify tradeoffs among functions; for example, it cannot distinguish between scenarios where two functions are performing at their extremes, indicative of strong tradeoffs, versus two functions performing at intermediate values, indicative of weaker tradeoffs^12^,^23^. For comparison with earlier studies, we conducted an analysis of multifunctionality using this averaging approach, and found a positive but saturating effect of species richness (Supplementary Fig. 10). A generalized log-linear mixed effects model predicted a 1% (±0.1) increase in average multifunctionality with each 10% increase in richness (*t*_1151_=7.37, *P*<0.001). For an example assemblage of 16 species, then, a loss of all but one species would equate to a 22% decrease in average multifunctionality. A related index based on the geometric versus arithmetic mean^30^ yielded nearly identical results (Supplementary Fig. 11). However, given the shortcomings outlined above^23^, we are reticent to rely on inferences from the averaging approach and instead emphasize the more nuanced inferences from the threshold approach.

### Conclusions

Overall, rigorous analysis of all available experimental evidence showed that high biodiversity generally sustains high levels of multifunctionality in ecosystems, even though some functions tend to be high when others are low. Moreover, this result was stronger for herbivores than for plants, and consistent across habitats. Finally, the effect of biodiversity was stronger as more functions were considered, implying that biodiversity is critical to highly multifunctional natural ecosystems. The implication is that prior analyses linking biodiversity to single functions like productivity may systematically underestimate the functional importance of changing biodiversity. Our study provides the most comprehensive evaluation of the biodiversity–multifunctionality relationship to date, and extends previous investigations that focused almost exclusively on plant biodiversity and productivity in temperate grasslands. Further, because we examined only manipulative experiments, we can infer a general causal relationship between biodiversity and multifunctionality versus some previous analyses, which used correlations drawn from observational data^22^. We also weighted functions equally in our analysis, as we had no *a priori* reason to value one function over another. However, future applications of this framework may wish to explicitly prioritize certain function(s) over others. We conclude that conserving biodiversity is a viable strategy in managing natural systems to ensure the provision of high levels of many ecosystem functions, which may translate to increased delivery of goods and services.

## Methods

### Data set

We revisited all 192 studies in the database from^6^,^38^ to assess whether the authors reported ≥2 independent measures of functioning. Criteria for inclusion in the above database can be found in (refs 2, 5). We identified 94 experiments that met this criterion, and from these extracted the means, variances, sample sizes, relevant metadata and species names for monocultures. All studies used in the analysis can be found in Supplementary Table 1, and the full data set is available as a supplement.

Whereas previous meta-analyses considered only functions relating to standing stock of the target organism or their resources and efficiency of resource use^2^,^3^,^4^,^5^, we considered any response that was relevant to the functioning of the experimental ecosystem. These included, for example, measures of nutrient flux and standing stock >1 trophic level from the manipulated taxa. However, this choice meant that not all responses were expected to be maximized by biodiversity. For example, standing stock of resources are predicted to be minimized with increasing biodiversity of consumers^34^,^39^. Opposing expectations may lead to multifunctionality indices that are confounded, that is, higher values are not always indicative of a positive biodiversity effect. To account for this discrepancy, we assigned an expected direction of the biodiversity effect to each response based on existing ecological theory and/or the authors' original presentation of the experiments^23^,^36^. If the expected direction for a function could not be adequately identified and defended based on the available evidence presented in the original publication and/or ecological theory that existed at the time of publication, we excluded that function from our database. Then, where functions were expected to be minimized with increasing biodiversity (that is, the expected direction was negative), we performed the following transformation^23^ such that the lower bound for all functions was set at 0:


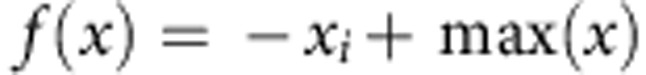


This transformation is analogous to the inversion of the log response ratios for functions relating to consumption used in other syntheses of biodiversity-ecosystem functioning^2^,^5^,^6^,^36^. These transformed values were carried through all subsequent analyses.

### Threshold approach

For each function in each experiment, we identified the maximum value of the mean response across all treatments. Next, we determined which treatments attained a given threshold (1–99%) of that maximum. Defining the maximum threshold at an unachievable high value, such as based on the single highest observed value, inevitably leads to finding no biodiversity effect at high thresholds because no biodiversity level can reach the threshold^23^. On the other hand, defining the maximum threshold at an easily achievable low value inevitably leads to finding no biodiversity effect at low thresholds because all biodiversity levels will reach the threshold. Our use of the maximum mean value may be lower than the maximum obtained if we took the average of the *n*-1 samples from an individual experiment, were we to have the raw data. Thus, care should be taken to avoid over-interpreting biodiversity effects near zero at high or low thresholds. We then tallied the number of functions that exceeded the given threshold for each level of richness. It is also important to note that we weighted functions equally in our analysis. Weighting of functions to reflect, for instance, management goals or prioritization of different functions is an exciting frontier, but we had no strong *a priori* reason to assign differential weighting to functions.

We modelled richness against the number of functions greater than a threshold using the following generalized mixed effects model to estimate the number of functions ≥ a threshold (*y*_*ij*_ observed, *μ*_*ij*_ estimated) at richness level *R*_*i*_ (*i*=1,…,Smax_*j*_) in study *j* with *F*_*j*_ numbers of functions measured. We assumed a quasipoisson error with a dispersion coefficient, *ω*.


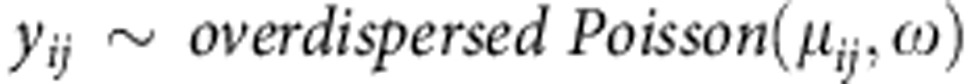



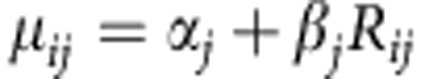


Where:


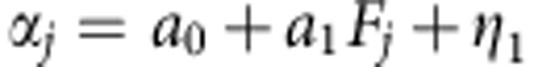



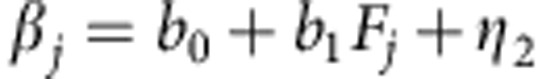



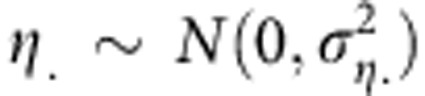


We included the number of functions measured in a study as a covariate to account for the mathematical effect it had on the maximum possible slope. This also allowed us to test whether the biodiversity effect changed based on the number of functions. We chose not to scale the response by the number of functions (to yield the proportion of functions greater than the threshold), as this implies that all relevant ecosystem functions had been measured. We fit this model to a quasipoisson distribution using an identity link, allowing the slope and intercept of the richness effect to vary by Study. We chose an identity link to aid in the interpretation of the regression coefficients (that is, the change in number of functions with addition of one species)^23^. We chose a quasi-likelihood approach to account for documented underdispersion in the data, particularly at low thresholds. We ran this model for every level of threshold, producing 99 models in total. We generated predicted fits, and extracted the pooled regression coefficients for the *richness*+*richness* × *number of functions* effect, and their pooled standard errors. We additionally extracted the coefficients for each level of the random factor to investigate the study-level variation in the biodiversity effect.

We also wanted to know whether this relationship changed as a function of the number of functions, habitat or trophic group. We thus fit a modified version of GLMM as above, including the effects of System (*S*_*j*_) and Trophic Group (*T*_*j*_), such that:


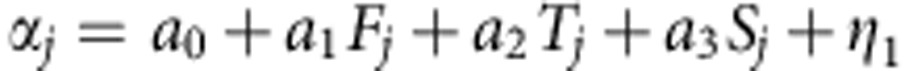



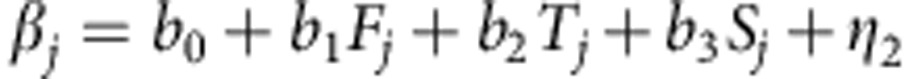


As above, we extracted the predicted fits for each level of the covariates.

### Turnover approach

To understand whether the identity of the best- and worst-performing species changed across multiple functions, we modified the index of turnover proposed in ref. 18. First, we identified the species that had the highest and lowest mean level of functioning in monoculture for each function in each experiment (adjusted for the expected direction). We then tallied the number of species necessary to maximize and minimize all functions in a given experiment, and divided this value by the total number of functions. Thus, the index represents the proportion of functions maximized or minimized by different species. We then averaged this index across studies to produce a mean and standard error.

### Simulation analysis

To understand the factors driving the observed dip in the biodiversity effect at intermediate thresholds, we designed a simulation to vary the proportion of functions *P* with a negative relationship to biodiversity. To begin, we identified two example studies that clearly exhibited the dip in our data set^40^,^41^. We extracted the following parameters from each: the maximum species richness *S*, the richness levels and the number of functions measured. For each treatment level and function, we then randomly generated values from a normal distribution *N*∼*S*,1. The mean was set at *S* so that there would initially be a positive, linear relationship of the responses with biodiversity. Then, some proportion of functions *P* were multiplied by −1 and their maximum added to reverse their relationship with biodiversity. We varied *P* from 0 to 1 in increments of 0.1. As with the main analysis, we calculated the number of functions above a threshold for each treatment, and fit a generalized linear model regressing this number against richness to a quasipoisson distribution using an identity link^23^. We repeated this procedure for all thresholds, from 1 to 99%, and extracted the linear coefficient for each threshold. Finally, we repeated this test 100 times for each of the two studies.

### Sensitivity analysis

We conducted a sensitivity analysis by individually removing each study from the data set and re-running the above threshold analyses. To reduce computation time, we selected 20, 40, 60 and 80% thresholds. From each threshold model, we extracted the regression coefficient corresponding to the richness effect. We then compared that coefficient to the coefficient from the corresponding threshold model run on the entire data set (Supplementary Fig. 12). In all cases, the 95% confidence intervals overlapped, indicating that no one study had an inordinate influence on our results.

### Averaging approach

To fairly combine responses measured on different scales, we transformed each function by dividing by the absolute value of the maximum observed level of functioning:


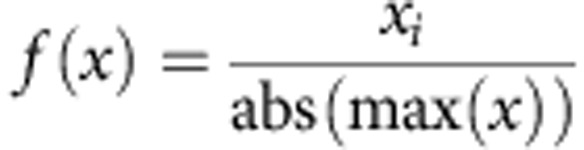


Here, *x* refers to the mean observed value of the function in biodiversity treatment *i*. This transformation put responses on the scale [0,1]. We derived an average index of multifunctionality by taking a mean of the scaled responses for each treatment in each experiment^23^.

We next modelled the effect of richness on average multifunctionality using a GLMM fit to a quasibinomial distribution with an identity link using the function *glmmPQL* in the MASS package^42^. Because we did not *a priori* expect average multifunctionality to have a linear relationship with biodiversity, we constructed models with different functional forms from^5^: null, linear, logarithmic, power and saturating (Michaelis–Menten)^2^,^5^. We then compared them using an AIC model selection approach^43^, which identified the log-linear model as the most parsimonious. We fit a log-linear model regressing our index of average multifunctionality (*y*_*ij*_) against richness (*R*_*ij*_), and allowed both the slope and intercept of the richness effect to vary by the random term of study:


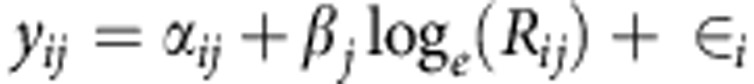



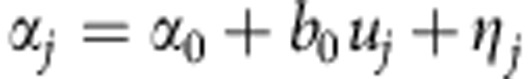



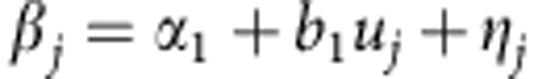


We extracted predicted fits and used the standard errors (s.e.) of the fixed effects to derive 95% confidence intervals (2*s.e.)^44^. We repeated this analysis using an alternative multiplicative index of multifunctionality that was the *n*th-root of the product of the scaled responses in each treatment (that is, the geometric mean)^30^.

To ascertain whether we arrived at conclusions regarding biodiversity's effect on multiple functions than on any single function, we performed a pooled analysis of single functions. We fit the same model as above, but included a nested effect of having measured a given function in a given study. As opposed to the average across functions, we set the scaled value of each individual function as the response. We found that there was virtually no difference in an analysis of average multifunctionality versus pooled single functions, which is actually not unexpected given that the average multifunctionality index is simply a mean of the individual functions^23^.

All analyses were conducted in R version 3.1.1^45^, and the R script used to conduct all analyses is included in Supplementary Note 1.

## Author Contributions

J.E.D., J.E.K.B., F.I., L.G., J.S.L., J.N.G. and B.J.C. conceived of the idea; J.S.L., J.E.D., J.E.K.B., F.I., L.G., J.G., N.E., M.J.S.H. and A.H. collected/contributed data; J.S.L. and J.E.K.B. analysed the data; J.S.L. drafted the paper with substantial input from all authors.

## Additional information

**How to cite this article:** Lefcheck, J. S. *et al*. Biodiversity enhances ecosystem multifunctionality across trophic levels and habitats. *Nat. Commun*. 6:6936 doi: 10.1038/ncomms7936 (2015).

## Supplementary Material

Supplementary Information Supplementary Figures 1-12, Supplementary Tables 1, Supplementary Note 1 and Supplementary References

Supplementary dataset 1 The full dataset used in the analysis, including (Tab 1) meta-data and (Tab 2) data.

## Figures and Tables

**Figure 1:**
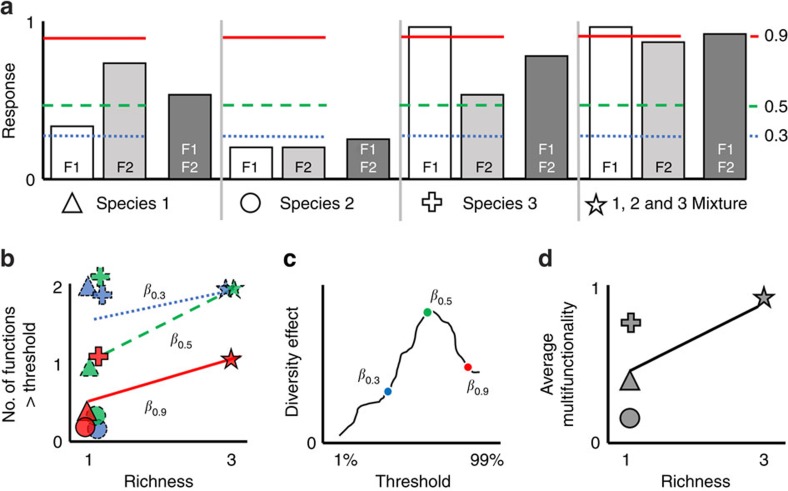
A conceptual illustration of the quantitative methods used to characterize multifunctionality. (**a**) A simple biodiversity experiment manipulating three species alone and all three in mixture, and measuring two ecosystem functions (F1 and F2) scaled between 0 and 1. Thresholds for 30%, 50% and 90% of the maximum observed level of functioning are denoted as the blue dotted, green dashed and red solid lines, respectively. A grand mean (dark grey bar) is taken across the two individual functions (white and light grey bars). (**b**) The number of functions exceeding a given threshold is plotted for each treatment, and regressed against richness. A positive slope (*β*) represents a positive effect of biodiversity on the number of functions exceeding a selected threshold. Line types correspond to threshold level in **a**. (**c**) The linear coefficients (*β*) from each regression in **c** are plotted against the corresponding threshold. In this case, all thresholds have been calculated from 1 to 99%, and the three coefficients from **b** are noted. (**d**) The grand mean from **a** is plotted for each treatment, and regressed against richness. A positive slope represents a positive effect of biodiversity on average multifunctionality.
