# Supplementary material for: Biodiversity enhances ecosystem multifunctionality across trophic levels and habitats
Source: Nat Commun. 2015 Apr 24;6:6936. doi: 10.1038/ncomms7936 (PMC4423209; doi:10.1038/ncomms7936)
Supplement: Supplementary Information — Supplementary Figures 1-12, Supplementary Tables 1, Supplementary Note 1 and Supplementary References [file ncomms7936-s1.pdf]

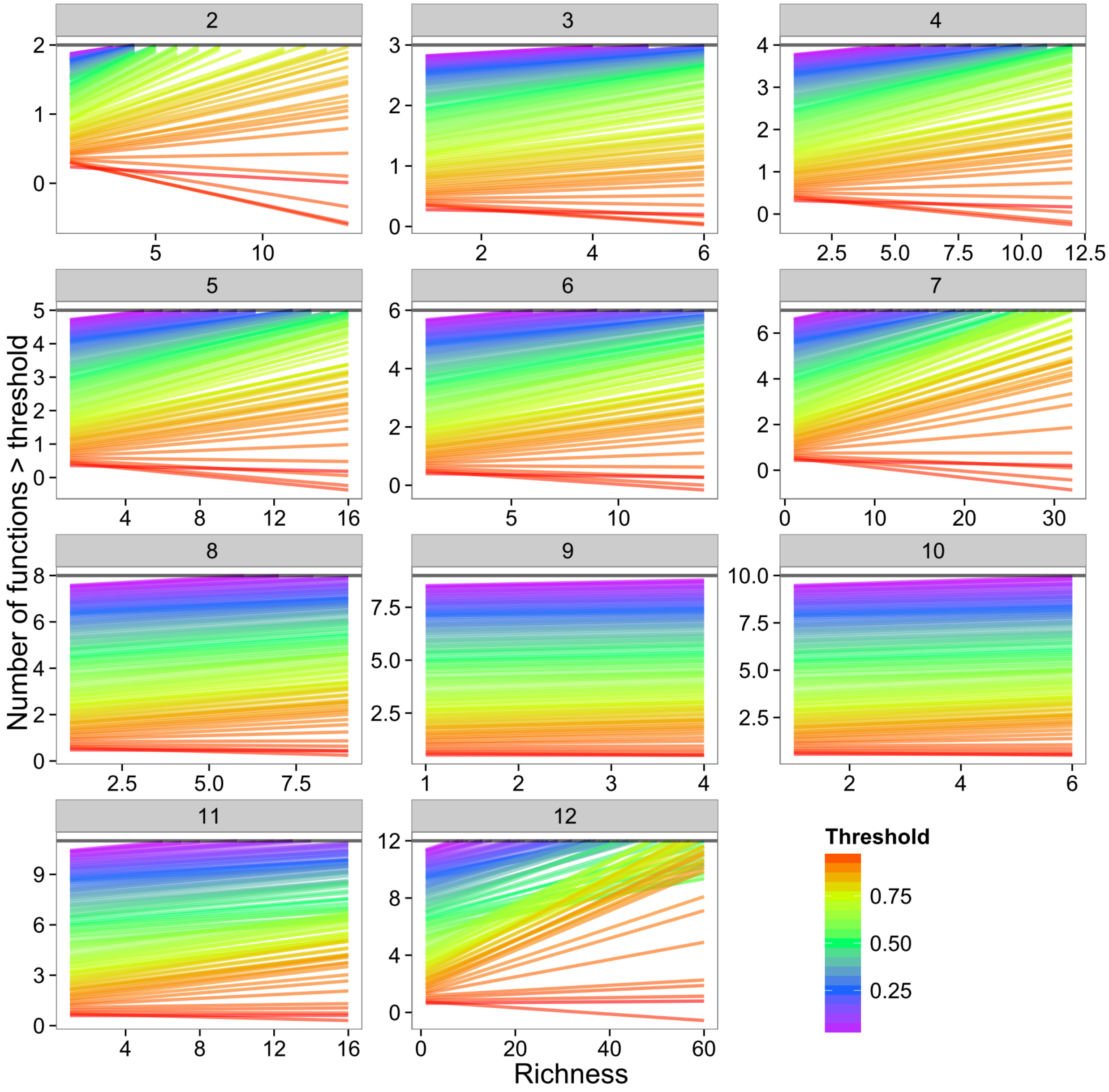

Supplementary Figure 1: The number of functions greater than a threshold against richness, for each level of number of functions (2-12). The raw number of functions greater than a threshold against richness, for thresholds ranging from 1% of the maximum (purple lines) to 99% of the maximum (red lines). Lines are predicted fits from generalized linear mixed effects models that fixed the covariate number of functions at the number indicated in the panel headers.

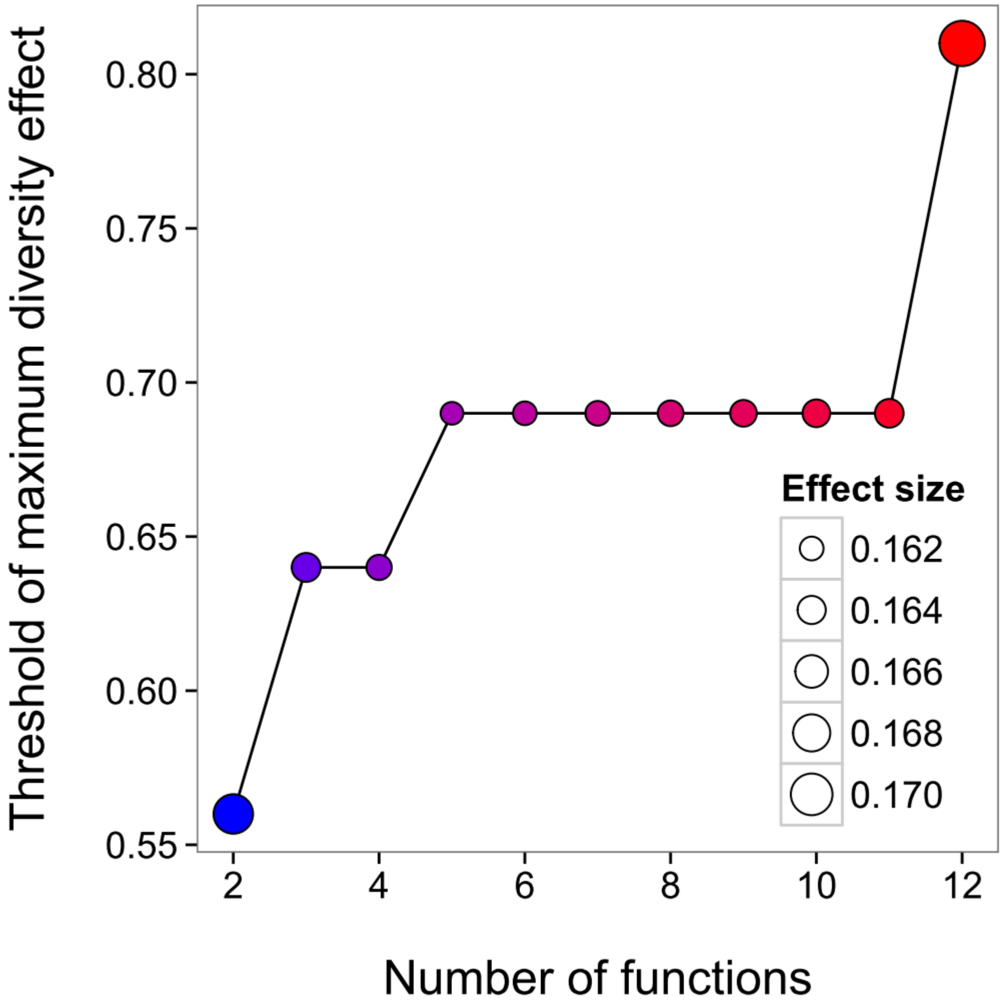

Supplementary Figure 2: The threshold where diversity has its maximum effect increased with the number of functions. The size of the points corresponds to the maximum effect size for each level of number of functions. Shading indicates the gradient from 2 functions (blue) to 12 functions (red).

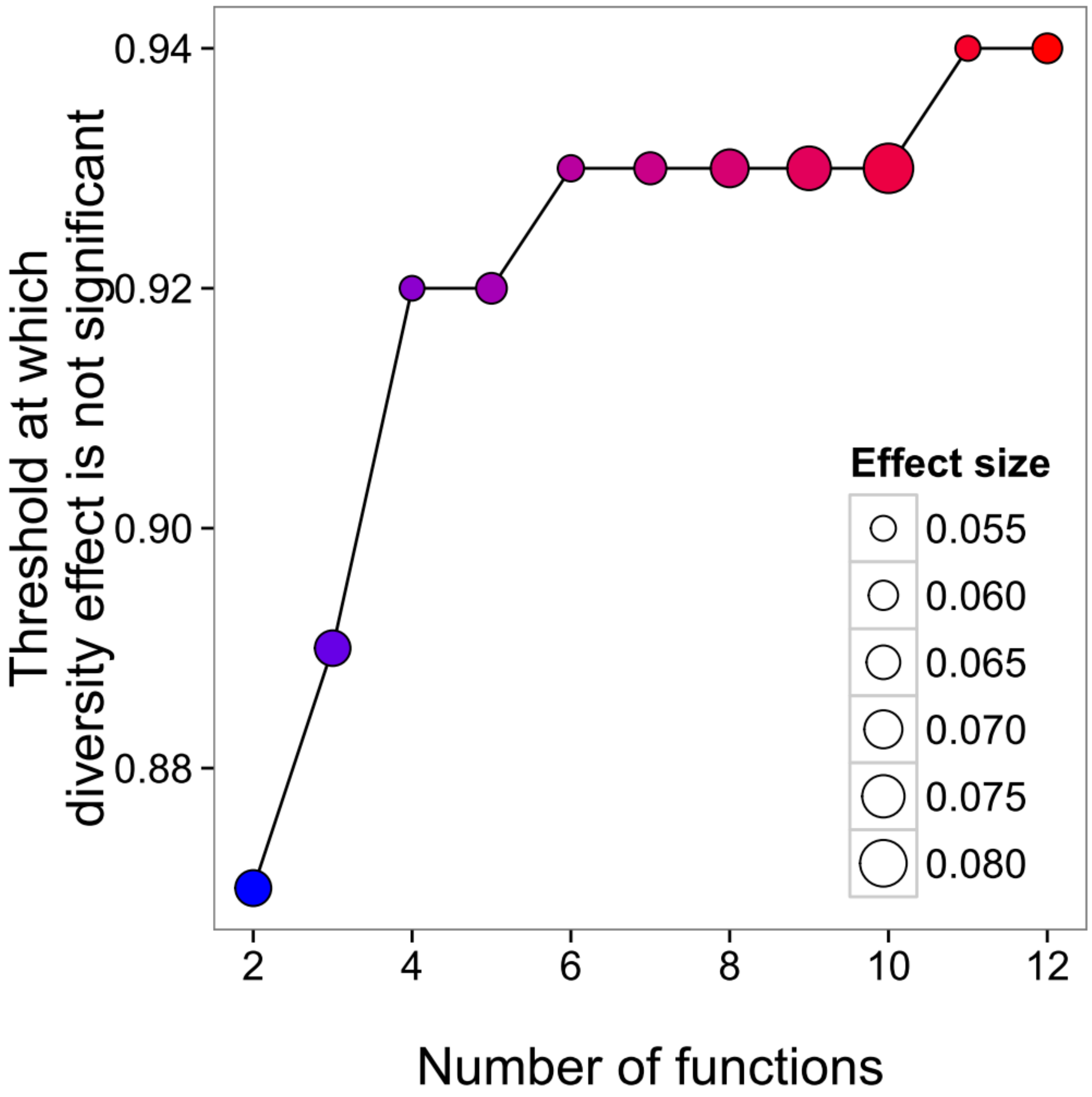

Supplementary Figure 3: The threshold after which diversity no longer has a significant effect increased with the number of functions. The size of the points corresponds to the effect size for each level of number of functions. Shading indicates the gradient from 2 functions (blue) to 12 functions (red).

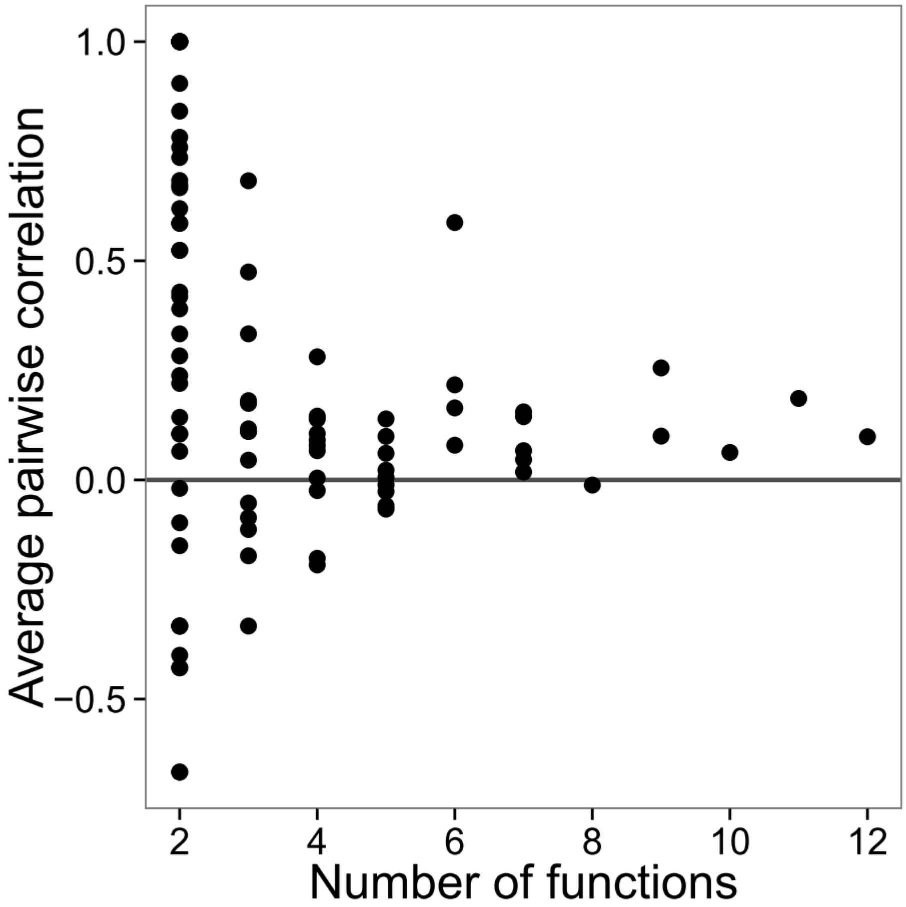

Supplementary Figure 4: The average pairwise correlation among all functions within a given experiment generally decreased with an increasing number of functions. Correlations were calculated as Kendall's rank correlations.

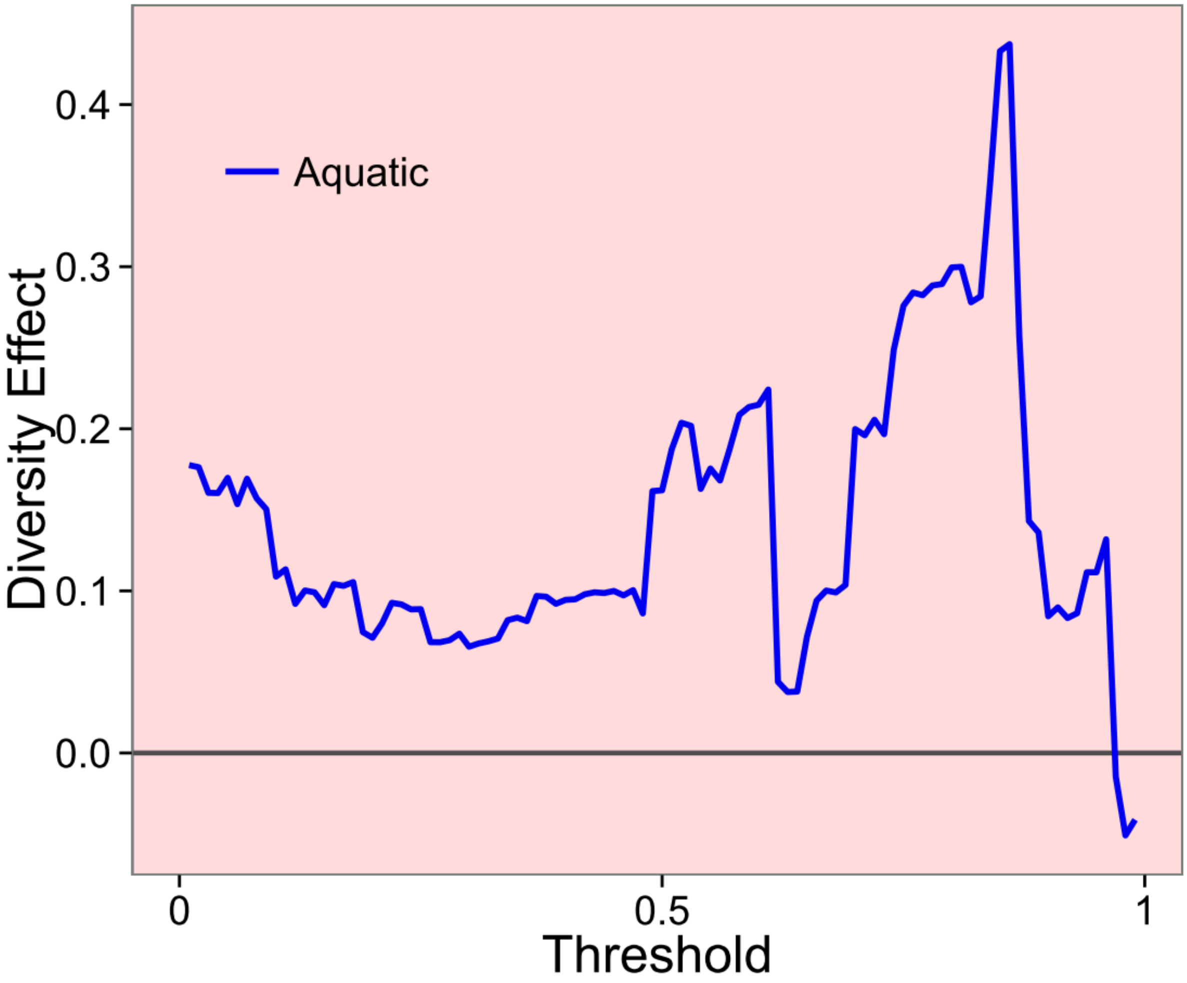

Supplementary Figure 5: The effect of diversity against threshold was highly variable for carnivores. The effect of biodiversity (linear coefficient regressing the number of functions above a threshold against richness) plotted against the continuum of thresholds from 1-99% of the maximum observed level of functioning. The blue line represents trends from experiments conducted in aquatic systems.

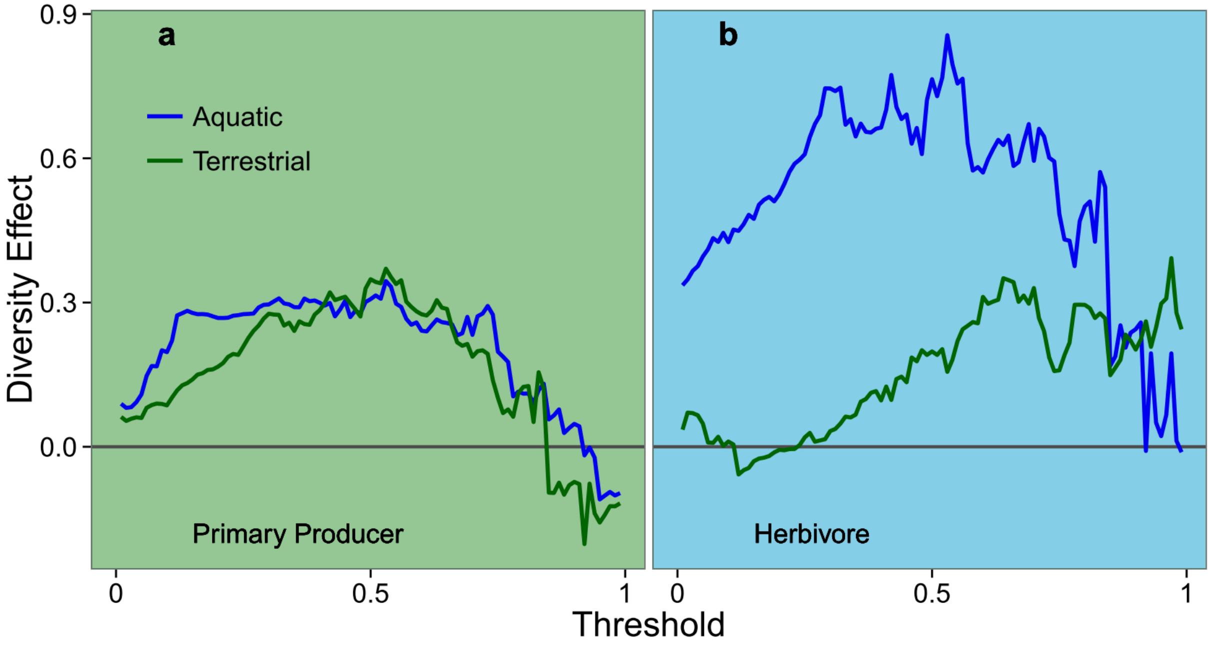

Supplementary Figure 6: Omitting all treatments where the number of species manipulated was *S* <= 6 revealed stronger effects for herbivores than for plants. The effect of biodiversity (linear coefficient regressing the number of functions above a threshold against richness) plotted against the continuum of thresholds from 1-99% of the maximum observed level of function for **(a)** plants, and **(b)** herbivores. Blue lines represent trends from experiments conducted in aquatic systems, and green lines from terrestrial systems.

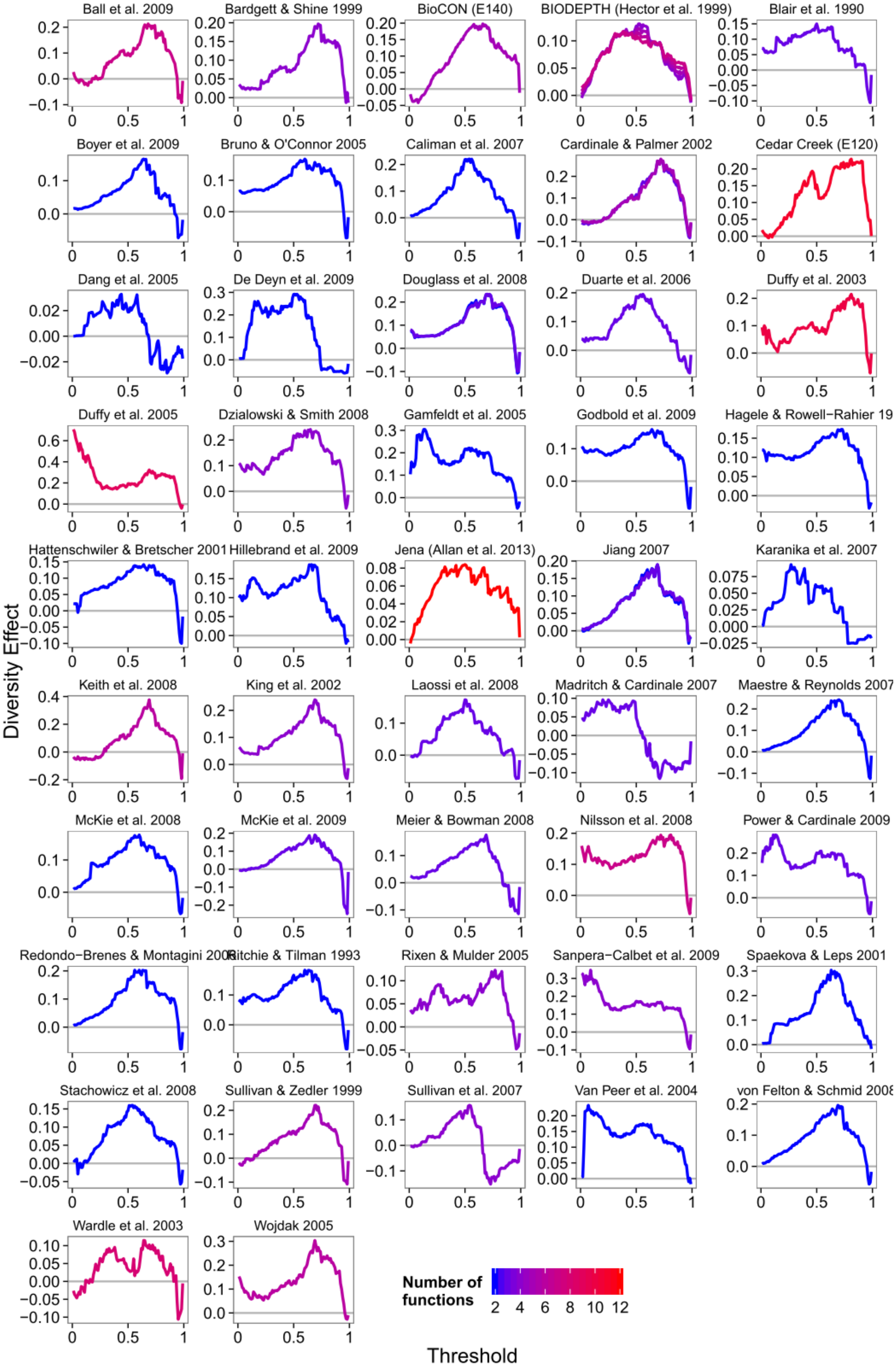
Ball et al. 2009
Bardgett & Shine 1999
BioCON (E140)
BIODEPTH (Hector et al. 1999)
Blair et al. 1990
Boyer et al. 2009
Bruno & O'Connor 2005
Caliman et al. 2007
Cardinale & Palmer 2002
Cedar Creek (E120)
Dang et al. 2005
De Deyn et al. 2009
Douglass et al. 2008
Duarte et al. 2006
Duffy et al. 2003
Duffy et al. 2005
Dzialowski & Smith 2008
Gamfeldt et al. 2005
Godbold et al. 2009
Hagele & Rowell−Rahier 19
Hattenschwiler & Bretscher 2001
Hillebrand et al. 2009
Jena (Allan et al. 2013)
Jiang 2007
Karanika et al. 2007
Keith et al. 2008
King et al. 2002
Laossi et al. 2008
Madritch & Cardinale 2007
Maestre & Reynolds 2007
McKie et al. 2008
McKie et al. 2009
Meier & Bowman 2008
Nilsson et al. 2008
Power & Cardinale 2009
Redondo−Brenes & Montagini 200
Ritchie & Tilman 1993
Rixen & Mulder 2005
Sanpera−Calbet et al. 2009
Spaekova & Leps 2001
Stachowicz et al. 2008
Sullivan & Zedler 1999
Sullivan et al. 2007
Van Peer et al. 2004
von Felton & Schmid 2008
Wardle et al. 2003
Wojdak 2005
Diversity Effect
Threshold
Number of functions
2 4 6 8 10 12

Supplementary Figure 7: Linear coefficients plotted against threshold for each study in the database. The effect of biodiversity (linear coefficient regressing the number of functions above a threshold against richness) plotted against the continuum of thresholds from 1-99% of the maximum observed level of functioning. Shading indicates the number of functions measured in each study from 2 (blue) to 12 (red). Panel headers correspond to studies used in the main analysis (see Supplementary Table 1).

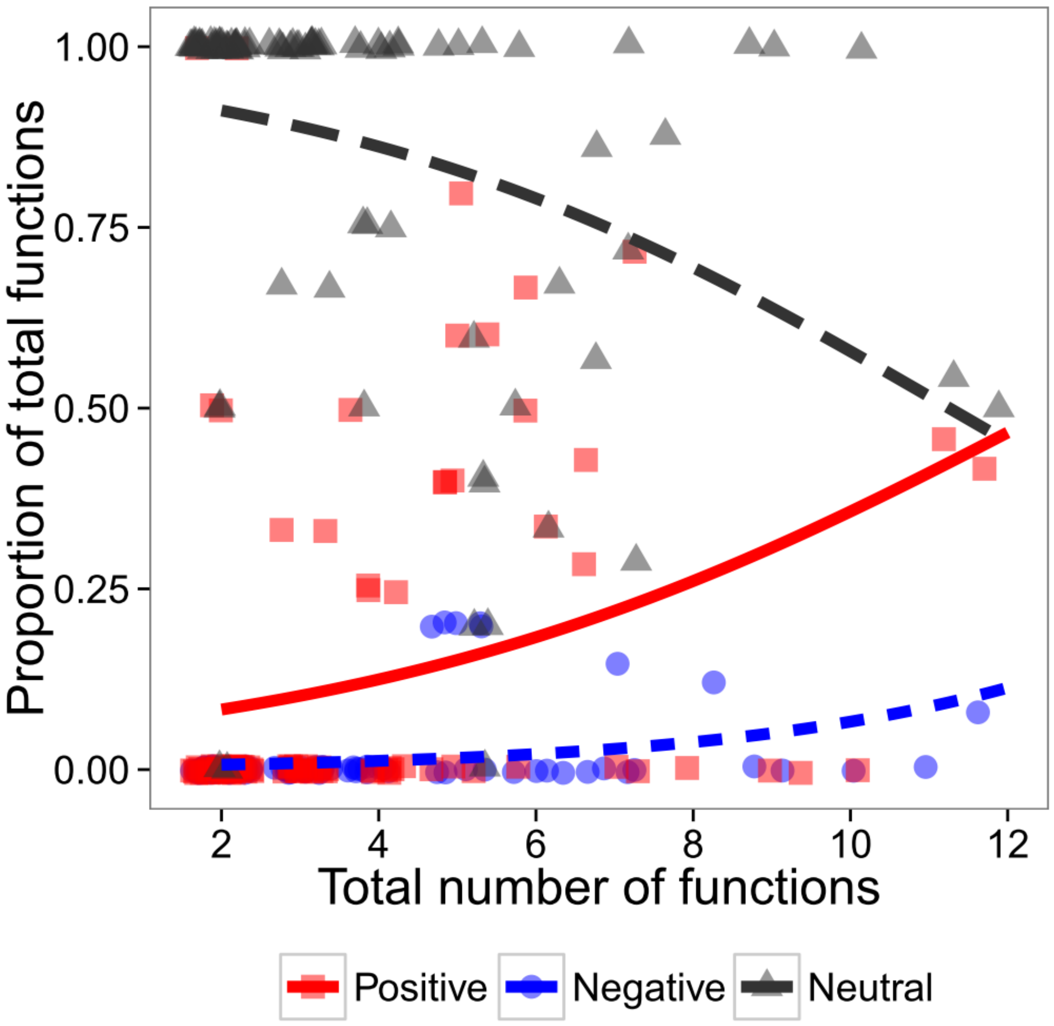

Supplementary Figure 8: The proportion of functions with a significantly positive relationship (solid red line) with diversity accumulated at a faster rate than those with significantly negative relationship (dotted blue line) in our dataset. The proportion of functions with a neutral (non-significant) relationship (dashed black line) also decreased with an increasing number of functions. Points are Kendell's rank correlations. Significance for each function was assessed using a test of association among paired samples ($\alpha = 0.05$). Lines represent fitted trends from a generalized linear model fit to a binomial distribution.

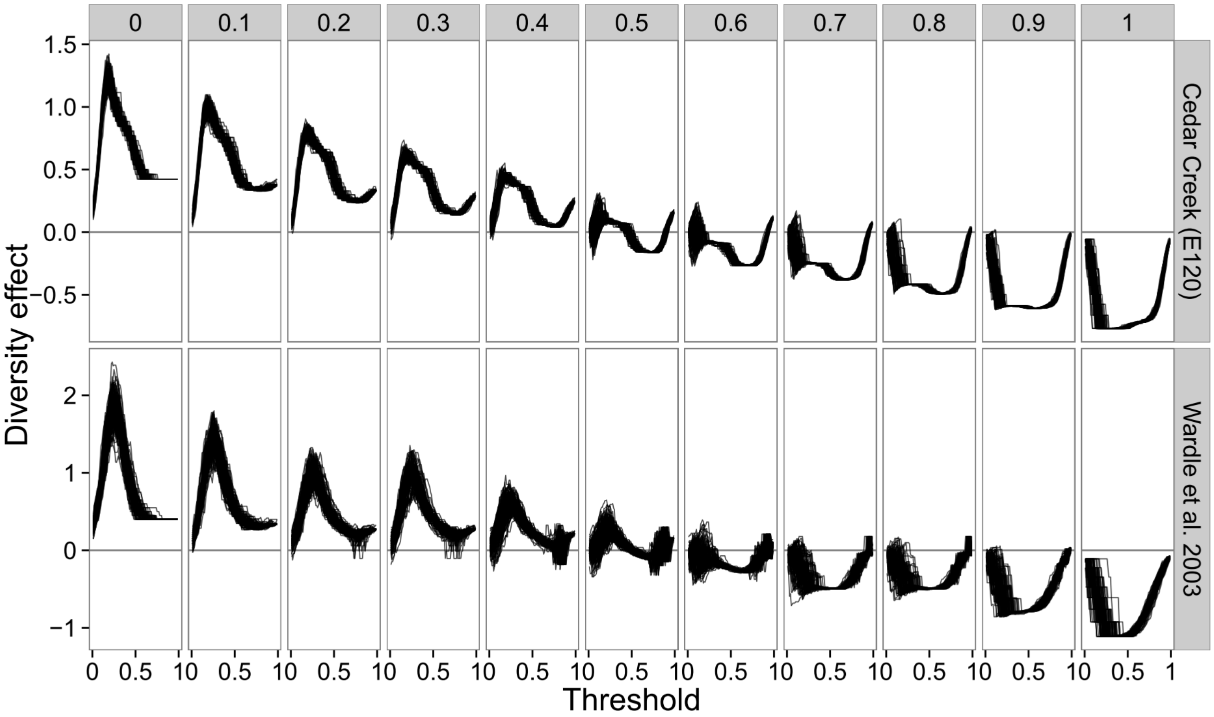

Supplementary Figure 9: Simulations showed that the distribution of diversity effects against threshold changed from concave-up to concave-down with an increasing proportion of negative effects (top panels, in increments of 0.1). Each line represents one of 100 runs of the simulation. The top row corresponds to parameters (richness, number of functions measured, diversity treatments) extracted from[1], and the bottom from[2].

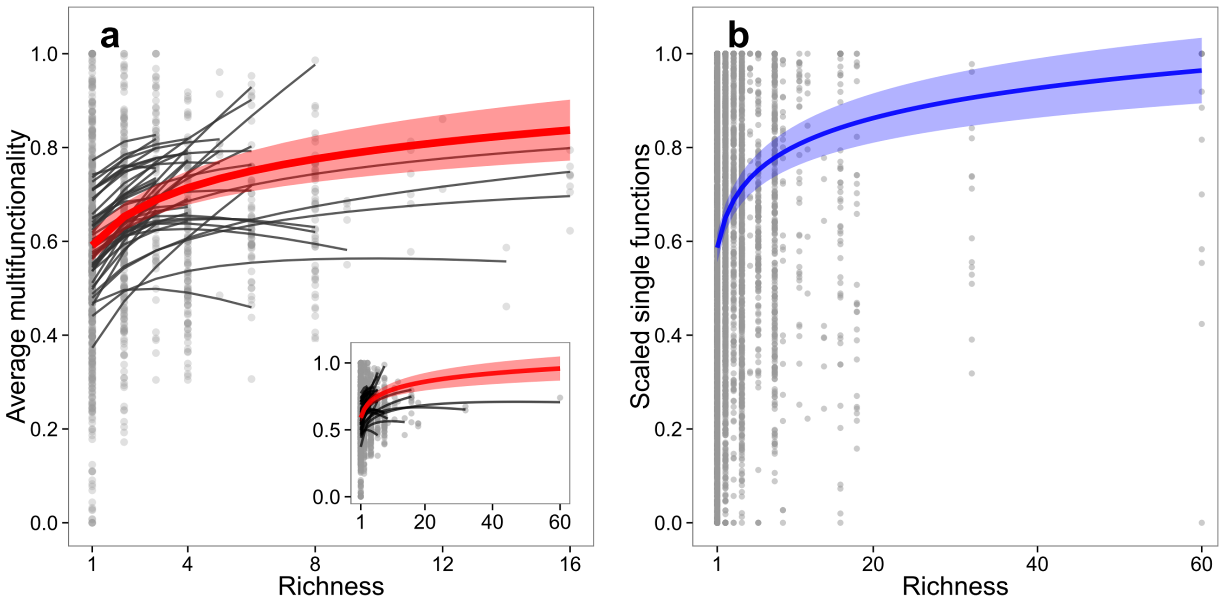

Supplementary Figure 10: Average multifunctionality showed a positive but decelerating relationship with richness. **(a)** The overall trend extracted from a generalized linear mixed effects model is given by the red line ± shaded 95% confidence intervals. Individual functions (points) and studies (black lines) varied in their response to increasing richness, but in many cases showed a similar trend. The main graphic shows the predicted trend for experiments that manipulated 16 or fewer species (96% of the dataset), while the inset shows this trend across all experiments in the dataset. **(b)** However, this trend was no different than a pooled analysis of single functions given by the blue line ± shaded 95% confidence intervals.

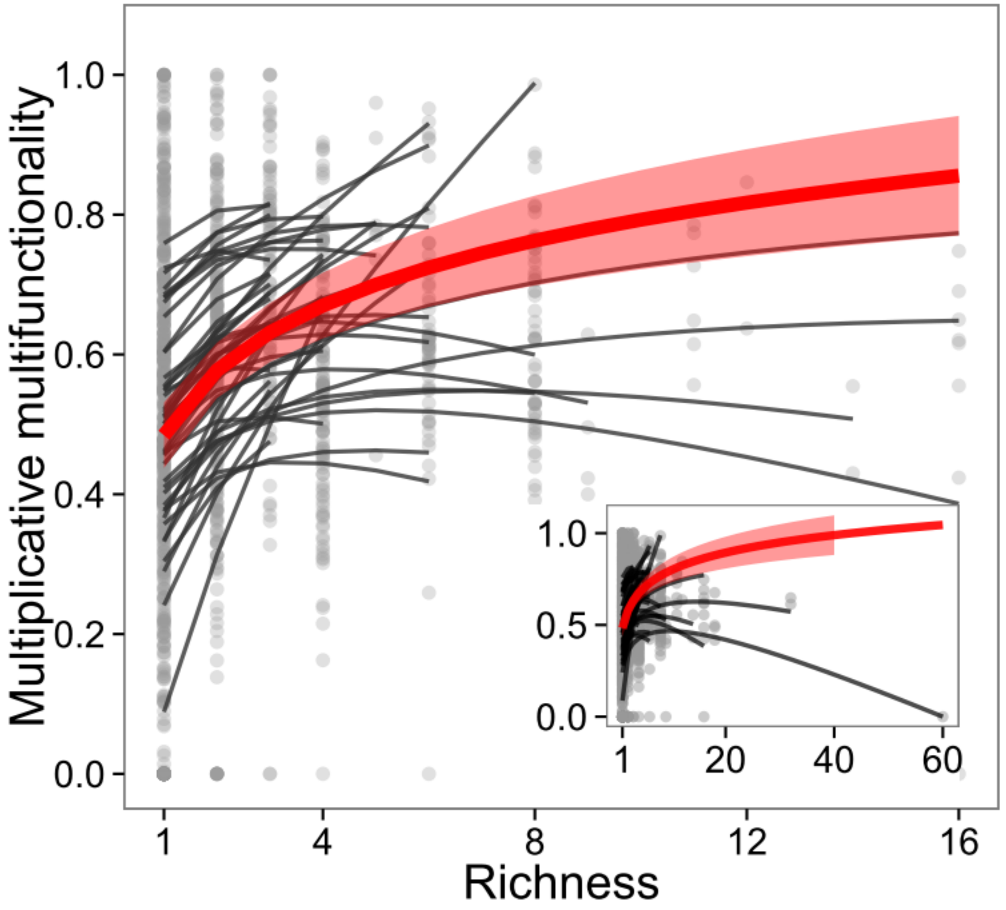

Supplementary Figure 11: Multiplicative multifunctionality showed a positive but decelerating relationship with richness. **(a)** The overall trend extracted from a generalized linear mixed effects model is given by the red line ± shaded 95% confidence intervals. Individual functions (points) and studies (black lines) varied in their response to increasing richness, but in many cases showed a similar trend. The main graphic shows the predicted trend for experiments that manipulated 16 or fewer species (96% of the dataset), while the inset shows this trend across all experiments in the dataset.

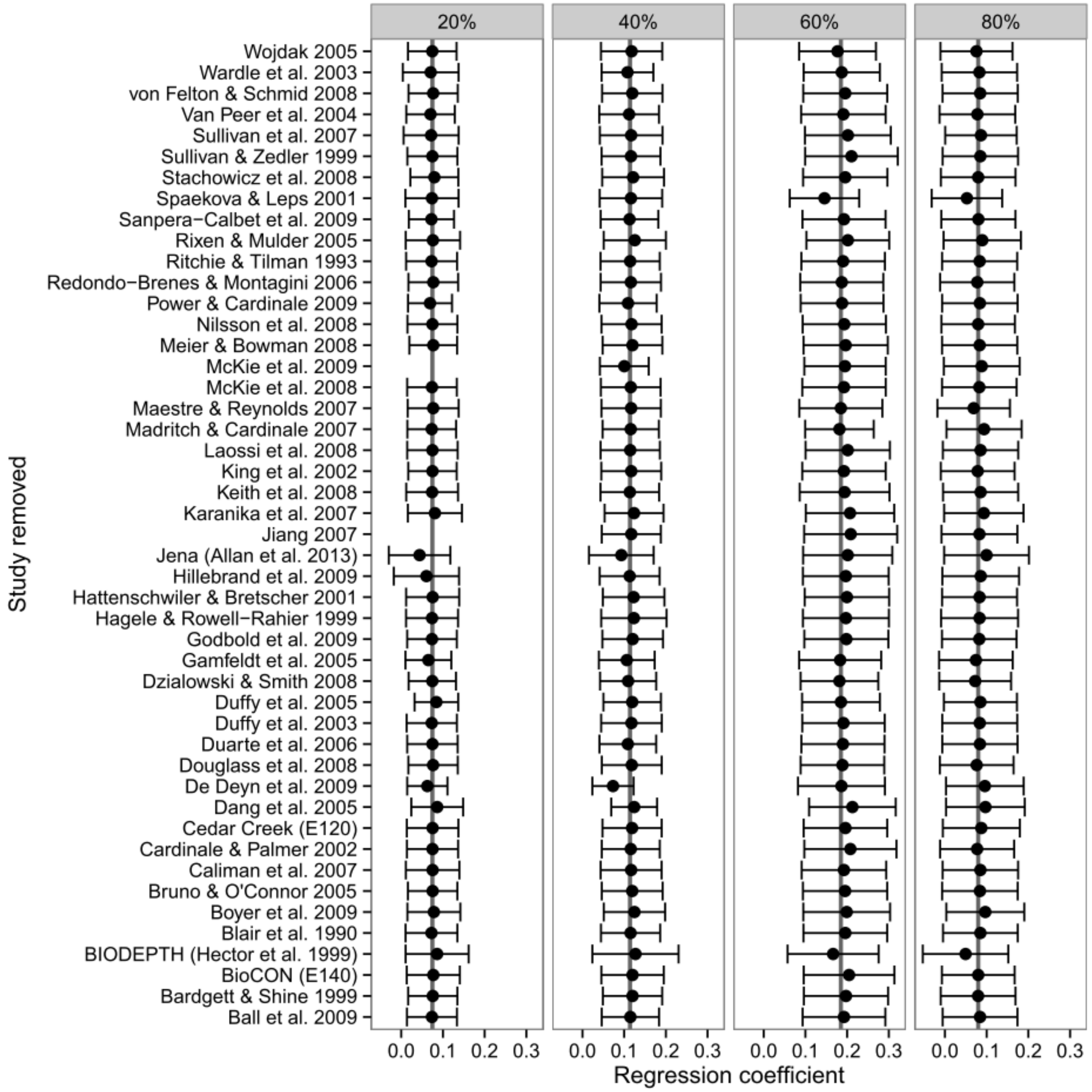

Supplementary Figure 12: Sensitivity analysis revealed that our results were robust to loss of any given study. Each point corresponds to the regression coefficient of the richness effect extracted from a generalized linear mixed effects model run on the reduced dataset missing the single study indicated on the y-axis. The error bars represent 95% confidence intervals, and the solid black line represents the overall regression coefficient obtained from a GLMM on the entire dataset. Each panel represents thresholds of 20, 40, 60, and 80% of the maximum.

Reference	Expt	System	Trophic Group	Max S	Max F	*r*
Ball et al. 2009 [3]	1	T	Dead organic matter	4	7	0.07
Bardgett & Shine 1999 [4]	1	T	Dead organic matter	6	4	0.09
BioCON (E140) [5]	1	T	Primary Producer	16	5	0.02
BioCON (E140) [5]	2	T	Primary Producer	16	5	0.14
BioCON (E140) [5]	3	T	Primary Producer	16	5	0.10
BioCON (E140) [5]	4	T	Primary Producer	16	5	0.01
BIODEPTH (Hector et al. 1999) [6]	1	T	Primary Producer	16	5	0.06
BIODEPTH (Hector et al. 1999) [6]	2	T	Primary Producer	18	7	0.14
BIODEPTH (Hector et al. 1999) [6]	3	T	Primary Producer	8	5	-0.01
BIODEPTH (Hector et al. 1999) [6]	4	T	Primary Producer	14	6	0.16
BIODEPTH (Hector et al. 1999) [6]	5	T	Primary Producer	12	4	0.14
BIODEPTH (Hector et al. 1999) [6]	6	T	Primary Producer	11	7	0.16
BIODEPTH (Hector et al. 1999) [6]	7	T	Primary Producer	12	6	0.22
BIODEPTH (Hector et al. 1999) [6]	8	T	Primary Producer	32	7	0.05
Blair et al. 1990 [7]	1	T	Dead organic matter	3	3	-0.33
Boyer et al. 2009 [8]	1	A	Primary Producer	6	2	0.39
Boyer et al. 2009 [8]	2	A	Primary Producer	6	2	0.62
Boyer et al. 2009 [8]	3	A	Primary Producer	6	2	-0.43
Boyer et al. 2009 [8]	4	A	Primary Producer	6	2	0.24
Bruno & O'Connor 2005 [9]	1	A	Carnivore	5	2	-0.15
Caliman et al. 2007 [10]	1	A	Detritivore	3	2	1.00
Caliman et al. 2007 [10]	2	A	Detritivore	3	2	1.00
Caliman et al. 2007 [10]	3	A	Detritivore	3	2	0.90
Cardinale & Palmer 2002 [11]	1	A	Detritivore	3	5	-0.06
Cardinale & Palmer 2002 [11]	2	A	Detritivore	3	3	0.18
Cedar Creek (E120) [1]	1	T	Primary Producer	17	11	0.19
Dang et al. 2005 [12]	1	A	Detritivore	8	2	-0.10
Dang et al. 2005 [12]	2	A	Detritivore	8	2	0.07
Dang et al. 2005 [12]	3	A	Detritivore	8	2	0.10
De Deyn et al. 2009 [13]	1	T	Primary Producer	6	2	0.74
De Deyn et al. 2009 [13]	2	T	Primary Producer	6	2	0.84
Douglass et al. 2008 [14]	1	A	Herbivore	3	2	-0.33
Douglass et al. 2008 [14]	2	A	Herbivore	3	3	0.11
Douglass et al. 2008 [14]	3	A	Herbivore	3	3	0.11
Douglass et al. 2008 [14]	4	A	Herbivore	3	3	0.11
Douglass et al. 2008 [14]	5	A	Carnivore	3	3	0.11
Duarte et al. 2006 [15]	1	A	Detritivore	4	3	0.11
Duffy et al. 2003 [16]	1	A	Herbivore	6	10	0.06
Duffy et al. 2005 [17]	1	A	Herbivore	4	9	0.26
Duffy et al. 2005 [17]	2	A	Herbivore	4	9	0.10
Dzialowski & Smith 2008 [18]	1	A	Herbivore	4	4	0.28
Dzialowski & Smith 2008 [18]	2	A	Herbivore	4	4	0.08
Gamfeldt et al. 2005 [19]	1	A	Herbivore	3	2	1.00

Gamfeldt et al. 2005 [19]	2	A	Primary Producer	3	2	0.67
Godbold et al. 2009 [20]	1	A	Herbivore	3	2	-0.67
Hagele & Rowell-Rahier 1999 [21]	1	T	Primary Producer	6	2	0.59
Hägele & Rowell-Rahier 1999 [21]	2	T	Primary Producer	6	2	0.14
Hägele & Rowell-Rahier 1999 [21]	3	T	Primary Producer	6	2	0.52
Hättenschwiler& Bretscher 2001 [22]	1	T	Dead organic matter	3	2	-0.33
Hättenschwiler& Bretscher 2001 [22]	2	T	Dead organic matter	3	2	-0.67
Hillebrand et al. 2009 [23]	1	A	Herbivore	6	2	0.28
Hillebrand et al. 2009 [23]	2	A	Herbivore	4	2	0.22
Jena Experiment [24]	1	T	Primary Producer	60	12	0.10
Jiang 2007 [25]	1	A	Detritivore	4	2	-0.02
Jiang 2007 [25]	2	A	Detritivore	4	3	-0.09
Karanika et al. 2007 [26]	1	T	Primary Producer	14	2	0.76
Keith et al. 2008 [27]	1	T	Dead organic matter	5	6	0.08
King et al. 2002 [28]	1	T	Dead organic matter	4	4	0.07
Laossi et al. 2008 [29]	1	T	Primary Producer	4	3	0.47
Madritch & Cardinale 2007 [30]	1	T	Dead organic matter	6	3	0.05
Madritch & Cardinale 2007 [30]	2	T	Dead organic matter	6	3	-0.11
Madritch & Cardinale 2007 [30]	3	T	Dead organic matter	6	3	-0.17
Maestre & Reynolds 2007 [31]	1	T	Primary Producer	3	2	0.52
Maestre & Reynolds 2007 [31]	2	T	Primary Producer	3	2	0.59
Maestre & Reynolds 2007 [31]	3	T	Primary Producer	3	2	0.68
Maestre & Reynolds 2007 [31]	4	T	Primary Producer	3	2	0.43
McKie et al. 2008 [32]	1	A	Detritivore	3	2	0.33
McKie et al. 2009 [33]	1	A	Detritivore	3	3	0.17
McKie et al. 2009 [33]	2	A	Detritivore	3	3	0.18
McKie et al. 2009 [33]	3	A	Detritivore	3	3	0.12
McKie et al. 2009 [33]	4	A	Detritivore	3	3	0.68
Meier & Bowman 2008 [34]	1	T	Dead organic matter	4	3	-0.05
Nilsson et al. 2008 [35]	1	A	Carnivore	3	7	0.02
Power & Cardinale 2009 [36]	1	A	Primary Producer	5	3	0.33
Redondo-Brenes & Montagini 2006 [37]	1	T	Primary Producer	3	2	1.00
Redondo-Brenes & Montagini 2006 [37]	2	T	Primary Producer	3	2	1.00
Redondo-Brenes & Montagini 2006 [37]	3	T	Primary Producer	3	2	1.00
Ritchie & Tilman 1993 [38]	1	T	Herbivore	3	2	-0.43
Ritchie & Tilman 1993 [38]	2	T	Herbivore	3	2	-0.33
Rixen & Mulder 2005 [39]	1	T	Primary Producer	8	4	0.00
Rixen & Mulder 2005 [39]	2	T	Primary Producer	8	4	-0.19
Rixen & Mulder 2005 [39]	3	T	Primary Producer	8	4	0.15
Rixen & Mulder 2005 [39]	4	T	Primary Producer	8	4	-0.02
Sanpera-Calbet et al. 2009 [40]	1	A	Dead organic matter	3	4	0.11
Spaekova & Leps 2001 [41]	1	T	Primary Producer	6	2	0.78
Spaekova & Leps 2001 [41]	2	T	Primary Producer	6	2	0.67
Stachowicz et al. 2008 [42]	1	A	Primary Producer	4	2	-0.40

Sullivan & Zedler 1999 [43]	1	T	Primary Producer	6	5	-0.03
Sullivan & Zedler 1999 [43]	2	T	Primary Producer	3	5	-0.07
Sullivan et al. 2007 [44]	1	T	Primary Producer	6	4	-0.18
Van Peer et al. 2004 [45]	1	T	Primary Producer	8	2	0.42
von Felten & Schmid 2008 [46]	1	T	Primary Producer	4	2	0.11
Wardle et al. 2003 [2]	1	T	Primary Producer	9	8	-0.01
Wojdak 2005 [47]	1	A	Herbivore	3	6	0.59

Supplementary Table 1: A list of all studies used in the analysis. *Expt* represents independent experiments conducted within the same study. *System* is either *A* = Aquatic or *T* = Terrestrial. *Max S* represents the maximum species richness manipulated. *Max F* represents the maximum number of functions recorded. The average correlation *r* is the mean of all pairwise rank correlations (Kendall's *r*) among functions in a given experiment.

Supplementary Note 1: R Code

```
##########################################################################################
#                                                                                        #
#                 META-ANALYSIS OF BIODIVERSITY AND ECOSYSTEM MULTIFUNCTIONALITY         #
#                                                                                        #
##########################################################################################

#Authors: Jon Lefcheck & Jarrett Byrnes

#Last updated: 2015-01-27

##########################################################################################
#                                       TABLE OF CONTENTS                                #
#   Line 23: Required libraries                                                          #
#   Line 32: Importing and formatting the data                                           #
#   Line 74: Data exploration                                                            #
#   Line 179: Multiple threshold approach                                                #
#   Line 554: Turnover approach                                                          #
#   Line 1198: Averaging approach                                                        #
#   Line 1247: Multiplicative approach                                                   #
#                                                                                        #
##########################################################################################

library(ggplot2) #Calls: ggplot
library(gridExtra) #Calls: grid.arrange
library(MASS) #Calls: glmmPQL
library(nlme) #Calls: lmeControl
library(plotrix) #Calls: std.error
library(plyr) #Calls: ddply, rbind.fill
library(reshape2) #Calls: melt

##########################################################################################
#                                IMPORTING AND FORMATTING THE DATA                       #
##########################################################################################

#Import from file: Monoculture meta-master ALL DATA.xlxs
multifunc=read.csv("Lefcheck et al Multifunc Meta.csv")

#Remove the rows where Direction!="Positive" or !="Negative"
multifunc=droplevels(subset(multifunc,multifunc$Direction=="Positive" | multifunc$Direction=="Negative"))

#Convert all response means, sample sizes, and standard deviations to numeric
#First, extract names of columns for response means, N, and SD
Y.colnames=colnames(multifunc)[ grep("Y",colnames(multifunc))[grep("Y",colnames(multifunc))>=27] ]
N.colnames=colnames(multifunc)[ grep("N",colnames(multifunc))[grep("N",colnames(multifunc))>=27] ]
SD.colnames=colnames(multifunc)[ grep("SD",colnames(multifunc))[grep("SD",colnames(multifunc))>=27] ]
#Convert response values to numeric
multifunc[,c(Y.colnames,N.colnames,SD.colnames)]=apply(multifunc[,c(Y.colnames,N.colnames,SD.colnames)],2,func
tion(x) as.numeric(as.character(x)) )

#Check recorded number of species (Smax) against actual number of species in maximum polyculture treatment
#First, retrieve the column name of the last column with actual values
poly.colnames=apply(multifunc[,Y.colnames],1,function(x) { y=rev(x[is.finite(x)])[1]; names(y)[length(y)] })
#Use regular exprsesions to grab the number in the column name and covert them to a numeric vector
Smax.colnames=as.numeric(gsub("X([0-9]+).*","\\1",poly.colnames))
cbind(as.character(multifunc$Reference),Smax.colnames,multifunc$Smax,Smax.colnames==multifunc$Smax)

#Check to see if any experiments report only one function
byexpt=ddply(multifunc,c("Reference","Study","Expt"),nrow)
byexpt[byexpt$V1==1,c("Reference","Study","Expt")]

#If Direction=="Negative", then transform based on Byrnes et al. 2014 MEE: x = -x + max(x)
multifunc[,Y.colnames]=ddply(multifunc,1,function(x)
  if(x$Direction=="Negative") -x[Y.colnames]+max(x[Y.colnames],na.rm=T) else x[Y.colnames] )[,-1]

#If any responses are negative, scale so that they are all >0
multifunc[,Y.colnames]=ddply(multifunc,1,function(x)
  if(any(x[Y.colnames]<0,na.rm=T)) x[Y.colnames]+max(abs(x[Y.colnames]),na.rm=T) else x[Y.colnames] )[,-1]

#Create a dataset with values scaled by the maximum value (for averaging approach)
multifunc.scaled=multifunc
multifunc.scaled[,SD.colnames]=ddply(multifunc,1,function(x) x[SD.colnames]/max(abs(x[Y.colnames]),na.rm=T)
)[,-1]
multifunc.scaled[,Y.colnames]=ddply(multifunc,1,function(x) x[Y.colnames]/max(abs(x[Y.colnames]),na.rm=T) )[,-
1]

##########################################################################################
#                                       DATA EXPLORATION                                 #
##########################################################################################

#Number of studies
nrow(ddply(multifunc,"Study",nrow))
#Number of experiments
nrow(ddply(multifunc,c("Study","Expt"),nrow))
```

```
#Total number of functions
nrow(multifunc)
#Number of experiments for each function
table(ddply(multifunc,c("Study","Expt"),nrow)$V1)

#Number of habitats
count(ddply(multifunc,c("Study","Expt","Sys1"),nrow),vars="Sys1")
#Number of trophic levels
count(ddply(multifunc,c("Study","Expt","FTG"),nrow),vars="FTG")
#And both
count(ddply(multifunc,c("Study","Expt","Sys1","FTG"),nrow),vars=c("FTG","Sys1"))

#Level of richness within an experiment
hist(multifunc$Smax)
median(multifunc$Smax)
range(multifunc$Smax)
#Level of richness within an experiment by habitat
ddply(multifunc,c("Sys1"),summarize,median=median(Smax))
ddply(multifunc,c("Sys1"),function(x) data.frame(min=range(x$Smax)[1],max=range(x$Smax)[2]))
#Level of richness within an experiment by trophic level
ddply(multifunc,c("FTG"),summarize,median=median(Smax))
ddply(multifunc,c("FTG"),function(x) data.frame(min=range(x$Smax)[1],max=range(x$Smax)[2]))

#Number of functions per experiment
median(ddply(multifunc,c("Study","Expt"),nrow)$V1)
range(ddply(multifunc,c("Study","Expt"),nrow)$V1)
#Number of functions per experiment by habitat
ddply(ddply(multifunc,c("Study","Expt","Sys1"),nrow),"Sys1",function(x) data.frame(median=median(x$V1)))
ddply(ddply(multifunc,c("Study","Expt","Sys1"),nrow),"Sys1",function(x)
data.frame(min=range(x$V1)[1],max=range(x$V1)[2]))
#Number of functions per experiment by trophic level
ddply(ddply(multifunc,c("Study","Expt","FTG"),nrow),"FTG",function(x) data.frame(median=median(x$V1)))
ddply(ddply(multifunc,c("Study","Expt","FTG"),nrow),"FTG",function(x)
data.frame(min=range(x$V1)[1],max=range(x$V1)[2]))

#Look at average pairwise correlation between all functions within a study
pairwisecor.df=ddply(multifunc,c("Reference","Study","Expt","Sys1","FTG"),function(x) {
  Smax=unique(x$Smax)
  x=x[,Y.colnames]
  cormat=cor(t(x),use="complete.obs",method=c("kendall"))
  data.frame(
    Smax=Smax,
    no.fn=nrow(x),
    avg.cor=mean(cormat[lower.tri(cormat)]) ) } ); pairwisecor.df
#And across all studies
mean(pairwisecor.df$avg.cor); std.error(pairwisecor.df$avg.cor)
#By habitat
ddply(pairwisecor.df,"Sys1",summarize,mean(avg.cor),std.error(avg.cor))
#By trophic level
ddply(pairwisecor.df,"FTG",summarize,mean(avg.cor),std.error(avg.cor))
#Plot as a function of number of functions
ggplot(pairwisecor.df,aes(x=no.fn,y=avg.cor))+
  geom_hline(yintercept=0,lwd=0.8,lty=1,col="grey30")+
  geom_point(size=3)+
  scale_x_continuous(breaks=seq(2,12,2))+
  labs(x="Number of functions",y="Average pairwise correlation")+
  theme_bw(base_size=18)+
  theme(panel.grid.major=element_blank(),panel.grid.minor=element_blank())

#Look at number of positive / negative relationships
#First cast data.frame longways
multifunc.long=melt(cbind(multifunc[,c(2:4,6:7,9,12)],multifunc[,Y.colnames]),id.vars=c(1:7),measure.vars=c(8:
112))
multifunc.long$richness=suppressWarnings(
  ifelse(grepl("mono",multifunc.long$variable),1,as.numeric(gsub("X([0-
9]+).*","\\1",multifunc.long$variable))) )

#Next calculate correlation between richness and functioning for each function
diversitycor.df=ddply(multifunc.long,c("Reference","Study","Expt","Sys1","FTG","Ydesc"),function(x)
  data.frame(cor=cor(x$richness,x$value,use="complete.obs",method="kendall"),
             p.value=cor.test(x$richness,x$value,na.action=na.omit,method="kendall")$p.value) )

#Calculate number of positive/negative functions for each study
propcor.df=ddply(diversitycor.df,c("Study","Expt","Reference"),function(x)
  cbind(no.fn=length(unique(x$Ydesc)),
        sig.neg=sum(x[x$p.value<=0.05,"cor"]<0),
        sig.pos=sum(x[x$p.value<=0.05,"cor"]>0),
        neutral=length(x[x$p.value>0.05,"cor"])) )

propcor.df=melt(propcor.df,id.vars=c(1:4),measure.vars=c(5:7))
propcor.df$variable=factor(propcor.df$variable,levels=c("sig.pos","sig.neg","neutral"))
levels(propcor.df$variable)=c("Positive","Negative","Neutral")
#Set symbols based on references in simulation, below
# propcor.df=adply(propcor.df,1,function(x) {
# if(x$Reference=="Wardle et al. 2003") "diamond" else
#   if(x$Reference=="Cedar Creek (E120)") "triangle" else "none" } )

#Plot results
```

```
ggplot(propcor.df,aes(x=no.fn,y=value/no.fn,group=variable,col=variable,shape=variable))+#,shape=V1))+
  geom_point(size=4,alpha=0.5,position="jitter")+
  scale_x_continuous(breaks=c(2,4,6,8,10,12))+
  scale_color_manual(values=c("red","blue","grey20"),name="")+
  scale_shape_manual(values=15:17,name="")+
  #   scale_shape_manual(values=c(15,1,17),guide="none")+
  stat_smooth(method="glm",family=binomial(),aes(lty=variable),lwd=2,se=F)+
  scale_linetype(guide="none")+
  labs(x="Total number of functions",y="Proportion of total functions")+
  theme_bw(base_size=18)+
  theme(panel.grid.major=element_blank(),panel.grid.minor=element_blank(),
        legend.direction="horizontal",legend.position="bottom")

#####################################################################################################
#                                  MULTIPLE THRESHOLD APPROACH                                      #
#####################################################################################################

#Create vector of thresholds from 1-99%
thresholds=(1:99)/100

#Loop through thresholds and studies to calculate number of treatments > threshold
thresholds.df=ldply(thresholds,.progress="text",function(thresh) {
  ddply(multifunc,c("Reference","Study","Expt"),function(x) {
    #Melt response values and relevant metadata
    responses=melt(cbind(x[,c(2:4,6:7,9,12)],x[,Y.colnames]),id.vars=c(1:7),measure.vars=c(8:112))
    #Remove NA values
    #responses=responses[!is.na(responses$value),]
    #Create a column for richness
    responses$richness=suppressWarnings(
      ifelse(grepl("mono",responses$variable),1,as.numeric(gsub("X([0-9]+).*","\\1",responses$variable))) )
    #Determine whether each response for each functions >= some percentage (threshold) of maximum
    responses=ddply(responses,"Ydesc",function(y) cbind(y,greater.than=y$value>=thresh*max(y$value,na.rm=T)) )
    #Summarize for each treatment
    ddply(responses,c("Study","Expt","Reference","Sys1","FTG","variable","richness"),function(z) {
      data.frame(
        threshold=thresh,
        no.fn=length(z$greater.than),
        no.fn.greater=sum(z$greater.than),
        prop.fn.greater=sum(z$greater.than)/length(z$greater.than) ) } ) } ) } )

#####################################################################################################

#Use mixed models to look at trends generally across all studies by fitting raw counts to
#quasipoisson distribution for each level of threshold
rawmods.list=dlply(thresholds.df,"threshold",.progress="text",function(i) {
  #Set lmeControl for certain thresholds
  if(i$threshold %in% c(0.02,0.99)) control=lmeControl(opt="optim",msTol=1e-6) else
    control=lmeControl(opt="optim")
  #Function to run models
  f=function(x) glmmPQL(no.fn.greater~richness*no.fn,random=~richness|Study,
                        family=quasipoisson(link="identity"),start=c(0.15,0.05,0.1,0.001),
                        control=control,
                        verbose=F,data=x)
  #Run model, return NA if model fails
  safef=failwith(NA,f)
  safef(i)
} )

#Extract predicted fit for muscle plot
#Modified from: http://glmm.wikidot.com/faq
predictraw.df=ldply(1:99,.progress="text",function(i) {
  ldply(2:12,function(j) {
    #Create dataframe for predicted values only for richness
    newdata=expand.grid(
      threshold=i/100,

richness=1:max(subset(thresholds.df,no.fn==j)[!is.na(subset(thresholds.df,no.fn==j)$no.fn.greater),"richness"]
),
      no.fn=j,
      no.fn.greater=NA)
    if(!"glmmPQL" %in% class(rawmods.list[[i]]))  {
      return(newdata)
    } else {
      #Generate predicted values from model
      newdata$no.fn.greater=predict(rawmods.list[[i]],newdata,type="response",level=0)
      #Return newdata
      return(newdata) }
  } ) } )

#Remove predictions that exceed the number of functions measured or drop below zero
predictraw.sub.df=ddply(predictraw.df,"no.fn",function(x) {
  x=subset(x,no.fn.greater<=x$no.fn & no.fn.greater>=0)
  data.frame(
    threshold=x$threshold,
    richness=x$richness,
    no.fn=x$no.fn,
    no.fn.greater=x$no.fn.greater)
} )
```

```
predictraw.sub.df=ddply(predictraw.df,"no.fn",function(x) subset(x,no.fn.greater<=no.fn))

#Plot predicted values against richness
ggplot(predictraw.sub.df,aes(x=richness,y=no.fn.greater,color=threshold,group=threshold))+
  geom_line(lwd=1,alpha=0.6)+
  geom_hline(aes(yintercept=no.fn),lwd=1,alpha=0.6,lty=1)+
  scale_color_gradientn(colours=rev(rainbow(5)),name="Threshold")+
  labs(x="Richness",y="Number of functions > threshold")+
  facet_wrap(~no.fn,scales="free",nrow=4)+
  theme_bw(base_size=18)+
  theme(panel.grid.major=element_blank(),panel.grid.minor=element_blank(),
        legend.position=c(0.8,0.1) )

#Break out no.fn==max(no.fn) for Figure 2 panel a
p1=ggplot(subset(predictraw.df,no.fn==12 & no.fn.greater<=12 &
no.fn.greater>=0),aes(x=richness,y=no.fn.greater,color=threshold,group=threshold))+
  #geom_hline(yintercept=1,lwd=0.8,lty=1,col="grey30")+
  geom_line(lwd=1,alpha=0.6)+
  geom_hline(yintercept=12,lwd=1,alpha=0.6,lty=1)+
  scale_color_gradientn(colours=rev(rainbow(5)),name="Threshold")+
  labs(x="Richness",y="Number of functions > threshold")+
  scale_x_continuous(breaks=c(1,20,40,60))+
  scale_y_continuous(limits=c(-0.1,14.5),breaks=c(0,4,8,12))+
  annotate("text",x=1,y=Inf,label="a",vjust=1.5,col="black",fontface="bold",size=7)+
  theme_bw(base_size=18)+
  guides(colour=guide_colourbar(title.position="top"))+
  theme(panel.grid.major=element_blank(),panel.grid.minor=element_blank(),legend.background=element_blank(),
        legend.position=c(0.7,0.9),legend.direction="horizontal",legend.box="horizontal"); p1

#Extract coefficients and standard errors
rawcoefs.df=ldply(2:max(thresholds.df$no.fn),.progress="text",function(i) {
  ldply(rawmods.list,function(j) {
    if("glmmPQL" %in% class(j)) {
      data.frame(
        no.fn=i,
        Intercept=summary(j)$tTable["(Intercept)","Value"]+i*summary(j)$tTable["no.fn","Value"],
        Intercept.Std.Error=sqrt(
(summary(j)$tTable["(Intercept)","Std.Error"]^2+summary(j)$tTable["no.fn","Std.Error"]^2+
                        2*j$varFix["(Intercept)","no.fn"]) ),
        Estimate=summary(j)$tTable["richness","Value"]+i*summary(j)$tTable["richness:no.fn","Value"],
        #Std.Error=summary(j)$tTable["richness","Std.Error"] )
        Estimate.Std.Error=sqrt(
(summary(j)$tTable["richness","Std.Error"]^2+summary(j)$tTable["richness:no.fn","Std.Error"]^2+
                       2*j$varFix["richness","richness:no.fn"]) ) )
    } else {
      data.frame(no.fn=i,Intercept=NA,Intercept.Std.Error=NA,Estimate=NA,Estimate.Std.Error=NA) }
  } ) } )
#Determine which thresholds are significantly different from zero
rawcoefs.df$sig=ifelse(rawcoefs.df$Estimate>2*rawcoefs.df$Estimate.Std.Error,"sig","not.sig")

#Extract threshold of max diversity effect & max threshold at which diversity has a positive effect
maxeffect.df=ddply(rawcoefs.df,"no.fn",function(x) {
  rbind(
    #Find threshold of maximum diversity effect
    cbind(type="max.div.effect",x[which.max(x$Estimate),]),
    #Find maximum threshold at which diversity has a significant positive effect
    cbind(type="max.threshold",x[rev(which(x$sig=="sig"))[1],]) ) } )

#Rename levels for plotting
#levels(maxeffect.df$type)=c("Threshold of maximum diversity effect","Maximum threshold where diversity effect
> 0")
maxeffect.df$type=factor(maxeffect.df$type,levels=c("max.div.effect","max.threshold"))

#Plot threshold against effect size with 95% confidence intervals
p2=ggplot(rawcoefs.df,aes(x=threshold,y=Estimate,group=no.fn))+
  geom_hline(xintercept=0,lwd=0.8,lty=1,col="grey70")+
  geom_ribbon(aes(fill=no.fn,ymax=Estimate+2*Estimate.Std.Error,ymin=Estimate-
2*Estimate.Std.Error),alpha=0.05)+
  scale_fill_gradient(high="red",low="blue",name="Number of\nfunctions")+
  geom_line(size=1,aes(col=no.fn))+
  scale_color_gradient(high="red",low="blue",name="Number of\nfunctions")+
  labs(x="Threshold",y="Diversity effect (Linear coefficients)")+
  geom_point(data=subset(maxeffect.df,no.fn %in% c(2,12)),
            aes(x=threshold,y=Estimate,shape=as.factor(threshold),fill=no.fn),col="white",size=8,width=2)+
  scale_shape_manual(values=c(21:24),guide=F)+
  annotate("text",x=0.01,y=Inf,label="b",vjust=1.5,col="black",fontface="bold",size=7)+
  theme_bw(base_size=18)+
  #guides(colour=guide_colourbar(title.position="top"))+
  theme(panel.grid.major=element_blank(),panel.grid.minor=element_blank(),
        legend.direction="horizontal",legend.box="horizontal",legend.position=c(0.275,0.1)); p2

#Generate Figure 2
grid.arrange(arrangeGrob(p1,p2,ncol=2,widths=c(1.5,2)))

#Look at how intercept changes as a function of threshold
ggplot(rawcoefs.df,aes(x=threshold,y=Intercept))+
  geom_ribbon(aes(fill=no.fn,ymax=Intercept+2*Intercept.Std.Error,ymin=Intercept-2*Intercept.Std.Error),
             alpha=0.2)+
```

```
  scale_fill_gradient(high="red",low="blue",name="Number of\nfunctions")+
  geom_line(size=1,aes(col=no.fn))+
  scale_color_gradient(high="red",low="blue",name="Number of\nfunctions")+
  facet_wrap(~no.fn,scales="free",nrow=2)+
  labs(x="\nThreshold",y="Intercept\n")+
  theme_bw(base_size=18)+
  theme(panel.grid.major=element_blank(),panel.grid.minor=element_blank(),legend.position="none")

#Plot against number of functions
ggplot(maxeffect.df,aes(x=no.fn,y=threshold,group=type,fill=no.fn))+
  #Add trend line
  geom_line()+
  #Scale points by the size of the diversity effect
  geom_point(aes(size=Estimate),shape=21)+

#geom_text(data=data.frame(type=c("max.threshold","max.div.effect"),no.fn=c(2.3,2.3),threshold=c(0.935,0.795),
lab=c("d","c")),
  #          aes(label=lab),size=8,fontface="bold")+
  #Break out panels by response
  facet_wrap(~type,ncol=1,scales="free_y")+
  scale_fill_gradient(high="red",low="blue",name="Number of\nfunctions")+
  scale_size(range=c(4,8),name="Effect size")+
  labs(x="\nNumber of functions",y="Threshold\n")+
  #Specify axis breaks
  scale_x_continuous(breaks=c(2,4,6,8,10,12))+
  theme_bw(base_size=18)+
  theme(panel.grid.major=element_blank(),panel.grid.minor=element_blank(),
        strip.background=element_blank(),strip.text=element_blank())

#Break out into separate plots
ggplot(subset(maxeffect.df,type=="max.div.effect"),aes(x=no.fn,y=threshold,fill=no.fn))+
  #Add trend line
  geom_line()+
  #Scale points by the size of the diversity effect
  geom_point(aes(size=Estimate),shape=21)+
  scale_fill_gradient(high="red",low="blue",name="Number of\nfunctions",guide=F)+
  scale_size(range=c(4,8),name="Effect size")+
  labs(x="\nNumber of functions",y="Threshold of maximum diversity effect\n")+
  #Specify axis breaks
  scale_x_continuous(breaks=c(2,4,6,8,10,12))+
  theme_bw(base_size=18)+
  theme(panel.grid.major=element_blank(),panel.grid.minor=element_blank(),
        strip.background=element_blank(),strip.text=element_blank(),
        legend.position=c(0.85,0.24))

ggplot(subset(maxeffect.df,type=="max.threshold"),aes(x=no.fn,y=threshold,fill=no.fn))+
  #Add trend line
  geom_line()+
  #Scale points by the size of the diversity effect #Scale points by the size of the diversity effect
  geom_point(aes(size=Estimate),shape=21)+
  scale_fill_gradient(high="red",low="blue",name="Number of\nfunctions",guide=F)+
  scale_size(range=c(4,8),name="Effect size")+
  labs(x="\nNumber of functions",y="Threshold at which\ndiversity effect is not significant")+
  #Specify axis breaks
  scale_x_continuous(breaks=c(2,4,6,8,10,12))+
  theme_bw(base_size=18)+
  theme(panel.grid.major=element_blank(),panel.grid.minor=element_blank(),
        strip.background=element_blank(),strip.text=element_blank(),
        legend.position=c(0.85,0.28))

####################################################################################################

#Extract slopes for each individual experiment from mixed model

#Create dataframe for predicted values for each study
predictindividual.df=ldply(1:99,.progress="text",function(i) {
  ldply(unique(thresholds.df$Study),function(j) {
    #Generate dataframe for predicted values
    newdata=expand.grid(
      Study=j,
      threshold=i/100,

richness=1:max(subset(thresholds.df,Study==j)[!is.na(subset(thresholds.df,Study==j)$no.fn.greater),"richness"]
),
      no.fn=max(subset(thresholds.df,Study==j)$no.fn))
    #Add predicted values with varying slope of richness for each study
    newdata$no.fn.greater=predict(rawmods.list[[i]],newdata,type="response")
    #Return dataframe
    return(newdata)
  } ) } )

#Add column for reference (for panel headers)
predictindividual.df$Reference=multifunc[match(predictindividual.df$Study,multifunc$Study),"Reference"]
#Generate muscle plots
ggplot(predictindividual.df,aes(x=richness,y=no.fn.greater,col=threshold,group=threshold))+
  geom_line(lwd=1,alpha=0.6)+
  scale_color_gradientn(colours=rev(rainbow(5)),name="Threshold")+
  labs(x="\nRichness",y="Number of functions > than threshold\n")+
```

```
  facet_wrap(~Reference,scales="free")+
  theme_bw(base_size=18)+
  theme(panel.grid.major=element_blank(),panel.grid.minor=element_blank(),strip.text.x=element_text(size=12),
        legend.position="right" )

#Extract coefficients for muscle plots
individualcoefs.df=ldply(1:99,.progress="text",function(i) {
  ldply(unique(thresholds.df$Study),function(j) {
    ldply(unique(thresholds.df[thresholds.df$Study==j,"no.fn"]),function(k) {
      data.frame(
        Study=rownames(coef(rawmods.list[[i]]))[j],
        no.fn=k,
        threshold=i/100,
        Intercept=coef(rawmods.list[[i]])[j,1]+k*coef(rawmods.list[[i]])[j,3],
        Estimate=coef(rawmods.list[[i]])[j,2]+k*coef(rawmods.list[[i]])[j,4] )
    } ) } ) } )

#Add column for reference (for panel headers)
individualcoefs.df$Reference=multifunc[match(individualcoefs.df$Study,multifunc$Study),"Reference"]
#Plot coefs against threshold
ggplot(individualcoefs.df,aes(x=threshold,y=Estimate,group=no.fn,col=no.fn))+
  geom_hline(xintercept=0,lwd=0.8,lty=1,col="grey70")+
  geom_line(lwd=1.2)+
  facet_wrap(~Reference,scales="free",ncol=5)+
  scale_color_gradient(high="red",low="blue",name="Number of\nfunctions")+
  scale_x_continuous(breaks=c(0,0.5,1),labels=c("0","0.5","1"))+
  labs(x="Threshold",y="Diversity Effect")+
  theme_bw(base_size=18)+
  theme(panel.grid.major=element_blank(),panel.grid.minor=element_blank(),
        strip.text.x=element_text(size=12),strip.background=element_blank(),
        legend.direction="horizontal",legend.position=c(0.55,0.025))
#Plot intercept against threshold
ggplot(individualcoefs.df,aes(x=threshold,y=Intercept,group=no.fn,col=no.fn))+
  geom_hline(xintercept=0,lwd=0.8,lty=1,col="grey70")+
  geom_line(lwd=1.2)+
  facet_wrap(~Reference,scales="free")+
  scale_color_gradient(high="red",low="blue",name="Number of\nfunctions")+
  labs(x="\nThreshold",y="Intercept\n")+
  theme_bw(base_size=18)+
  theme(panel.grid.major=element_blank(),panel.grid.minor=element_blank(),strip.text.x=element_text(size=12))

####################################################################################################

#Explore whether this relationship changes as a function of system or trophic level
thresholds.df$FTG=relevel(thresholds.df$FTG,"Primary Producer")
globalmods.list=dlply(thresholds.df,"threshold",.progress="text",function(i) {
  #Set lmeControl for certain thresholds
  if(i$threshold %in% c(0.46)) control=lmeControl() else
    control=lmeControl(opt="optim",msTol=1e-6)
  #Function to run models
  f=function(x) glmmPQL(no.fn.greater~richness*no.fn+richness*Sys1+richness*FTG,random=~richness|Study,
                        family=quasipoisson(link="identity"),
                        start=c(1,0.1,0.5,rep(0,11)),
                        control=control,
                        verbose=F,data=x)
  safef=failwith(NA,f)
  safef(i)
} )

#Create dataframe for predicted values for each system
predictglobal.df=ldply(1:99,.progress="text",function(i) {
  ldply(unique(thresholds.df$Sys1),function(j) {
    ldply(unique(thresholds.df$FTG),function(k) {
      #Subset dataframe
      data=subset(thresholds.df,Sys1==j & FTG==k)
      data=data[!is.na(data$no.fn.greater),]
      if("glmmPQL" %in% class(globalmods.list[[i]]))  {
        if(nrow(data)==0) {
          data.frame()
        } else {
          #Create new dataframe to store predictions
          newdata=expand.grid(
            Sys1=j,
            FTG=k,
            threshold=i/100,
            richness=1:max(data$richness),
            no.fn=max(data$no.fn))
          #Generate predicted values
          newdata$no.fn.greater=predict(globalmods.list[[i]],newdata,type="response",level=0)
          return(newdata) }
      } else {
        data.frame()
      } } ) } ) } )

#Plot predicted results by trophic group and system
ggplot(predictglobal.df,aes(x=richness,y=no.fn.greater,col=threshold,group=threshold))+
  geom_line(lwd=1,alpha=0.6)+
  scale_color_gradientn(colours=rev(rainbow(5)),name="Threshold")+
```

```
  labs(x="\nRichness",y="Number of functions > than threshold\n")+
  facet_wrap(Sys1~FTG,scales="free",nrow=2)+
  theme_bw(base_size=18)+
  theme(panel.grid.major=element_blank(),panel.grid.minor=element_blank(),
        legend.position="right" )

#Extract coefficients for muscle plots
globalcoefs.df=ldply(1:99,.progress="text",function(i) {
  ldply(unique(thresholds.df$Sys1),function(k) {
    ldply(unique(thresholds.df$FTG),function(l) {
      data=subset(thresholds.df,Sys1==k & FTG==l)
      if(nrow(data)==0) data.frame() else {
        ldply(2:max(data$no.fn),function(j) {
          if("glmmPQL" %in% class(globalmods.list[[i]])) {
            mod=globalmods.list[[i]]
            data.frame(
              threshold=i/100,
              Sys1=k,
              FTG=l,
              no.fn=j,
              Intercept=
                summary(mod)$tTable["(Intercept)","Value"]+
                ifelse(k=="Aquatic",0,summary(mod)$tTable[paste("Sys1",k,sep=""),"Value"])+
                ifelse(l=="Primary Producer",0,summary(mod)$tTable[paste("FTG",l,sep=""),"Value"])+
                j*summary(mod)$tTable["no.fn","Value"],
              Estimate=
                summary(mod)$tTable["richness","Value"]+
                ifelse(k=="Aquatic",0,summary(mod)$tTable[paste("richness:Sys1",k,sep=""),"Value"])+
                ifelse(l=="Primary Producer",0,summary(mod)$tTable[paste("richness:FTG",l,sep=""),"Value"])+
                j*summary(mod)$tTable["richness:no.fn","Value"] )
          } else {
            data.frame() }
        } ) } } ) } ) } )

globalcoefs.df$FTG=factor(globalcoefs.df$FTG,levels=c("Dead organic matter","Detritivore","Primary Producer",
                                                       "Herbivore","Carnivore"))
levels(globalcoefs.df$FTG)=c("Dead\norganic matter","Detritivore","Primary\nproducer","Herbivore","Carnivore")

#Plot coefs against threshold (subset out max number of functions): 7" x 7"
ggplot(ddply(subset(globalcoefs.df,FTG!="Carnivore"),c("Sys1","FTG"),function(x) subset(x,no.fn==max(no.fn))),
       aes(x=threshold,y=Estimate,col=Sys1,group=Sys1))+
  geom_rect(data=data.frame(
    FTG=levels(globalcoefs.df$FTG)[-5],
    Sys1=rep(levels(globalcoefs.df$Sys1),each=4),
    threshold=0,Estimate=0),
    aes(fill=FTG),xmin=-Inf,xmax=Inf,ymin=-Inf,ymax=Inf,alpha=0.15,show_guide=F)+
  scale_fill_manual(values=c("darkorange4","grey30","deepskyblue3","forestgreen"),guide="none")+
  geom_hline(xintercept=0,lwd=0.8,lty=1,col="grey30")+
  geom_line(lwd=1)+#aes(lty=Sys1),lwd=1)+
  #scale_linetype_manual(values=c(1,6))+
  scale_color_manual(values=c("blue2","darkgreen"),guide=guide_legend(ncol=1),name="")+
  facet_wrap(~FTG,nrow=2)+
  scale_x_continuous(breaks=c(0,0.5,1),labels=c("0","0.5","1"))+
  geom_text(data=data.frame(
    FTG=levels(globalcoefs.df$FTG)[-5],
    Sys1=rep(levels(globalcoefs.df$Sys1),each=4),
    labels=letters[1:4]),
    aes(x=0.1,y=Inf,label=labels),vjust=1.5,col="black",fontface="bold",size=6)+
  geom_text(data=data.frame(
    FTG=levels(globalcoefs.df$FTG)[-5],
    Sys1=rep(levels(globalcoefs.df$Sys1),each=4)),
    aes(x=0.15,y=-0.2,label=FTG),vjust=0,hjust=0,col="black",size=5)+
  labs(x="Threshold",y="Diversity Effect")+
  theme_bw(base_size=18)+
  theme(panel.grid.major=element_blank(),panel.grid.minor=element_blank(),
        strip.text.x=element_blank(),strip.background=element_blank(),
        legend.background=element_blank(),legend.key=element_blank(),
        legend.direction="horizontal",legend.box="horizontal",legend.position=c(0.87,0.9))

#Repeat but just for carnivores: 7" x 6"
ggplot(ddply(subset(globalcoefs.df,FTG=="Carnivore"),c("Sys1","FTG"),function(x) subset(x,no.fn==max(no.fn))),
       aes(x=threshold,y=Estimate,col=Sys1,group=Sys1))+
  geom_rect(data=data.frame(
    FTG=levels(globalcoefs.df$FTG)[-5],
    Sys1=rep(levels(globalcoefs.df$Sys1),each=4),
    threshold=0,Estimate=0),
    fill="firebrick1",xmin=-Inf,xmax=Inf,ymin=-Inf,ymax=Inf,alpha=0.025,show_guide=F)+
  geom_hline(xintercept=0,lwd=0.8,lty=1,col="grey30")+
  geom_line(lwd=1)+#aes(lty=Sys1),lwd=1)+
  #scale_linetype_manual(values=c(1,6))+
  scale_color_manual(values=c("blue2","darkgreen"),guide=guide_legend(ncol=1),name="")+
  scale_x_continuous(breaks=c(0,0.5,1),labels=c("0","0.5","1"))+
  #   geom_text(data=data.frame(
  #     FTG=levels(globalcoefs.df$FTG)[5],
  #     Sys1=rep(levels(globalcoefs.df$Sys1),each=1)),
  #     aes(x=0.15,y=-0.2,label=FTG),vjust=0,hjust=0,col="black",size=5)+
  labs(x="Threshold",y="Diversity Effect")+
  theme_bw(base_size=18)+
```

```
  theme(panel.grid.major=element_blank(),panel.grid.minor=element_blank(),
        strip.text.x=element_blank(),strip.background=element_blank(),
        legend.background=element_blank(),legend.key=element_blank(),
        legend.direction="horizontal",legend.box="horizontal",legend.position=c(0.2,0.77))

#Plot intercepts against threshold
ggplot(globalcoefs.df,aes(x=threshold,y=Intercept,col=no.fn,group=no.fn))+
  geom_hline(xintercept=0,lwd=0.8,lty=1,col="grey70")+
  geom_line(lwd=1.2)+
  scale_color_gradient(high="red",low="blue",name="Number of\nfunctions")+
  facet_grid(Sys1~FTG,scales="free")+
  scale_x_continuous(breaks=c(0,0.25,0.5,0.75,1))+
  labs(x="\nThreshold",y="Intercept\n")+
  theme_bw(base_size=18)+
  theme(panel.grid.major=element_blank(),panel.grid.minor=element_blank(),
        strip.text.x=element_text(size=12),legend.position=c(0.075,0.25) )

####################################################################################################

#Look at sensitivity of trophic levels results to Smax
#Identify studies with Smax > 6
ref.remove=unique(
  c(as.character(multifunc[multifunc$Smax > 6,"Reference"]),
    as.character(multifunc[!multifunc$FTG %in% c("Primary Producer","Herbivore"),"Reference"])) )
#Subset thresholds.sub.df to remove all experiments that manipulated Smax > 6
thresholds.sub.df=subset(thresholds.df,!Reference %in% ref.remove)
thresholds.sub.df$FTG=relevel(thresholds.sub.df$FTG,"Primary Producer")
#Re-run GLMMs
globalmods.sub.list=dlply(thresholds.sub.df,"threshold",.progress="text",function(i) {
  #Function to run models
  f=function(x) glmmPQL(no.fn.greater~richness*no.fn+richness*Sys1+richness*FTG,random=~richness|Study,
                        family=quasipoisson(link="identity"),
                        start=c(1,0.1,0.5,rep(0,5)),
                        control=lmeControl(opt="optim",msTol=1e-4),
                        verbose=F,data=x)
  safef=failwith(NA,f)
  safef(i)
} )

#Extract coefficients for muscle plots
globalcoefs.sub.df=ldply(1:99,.progress="text",function(i) {
  ldply(unique(thresholds.sub.df$Sys1),function(k) {
    ldply(unique(thresholds.sub.df$FTG),function(l) {
      data=subset(thresholds.sub.df,Sys1==k & FTG==l)
      if(nrow(data)==0) data.frame() else {
        ldply(2:max(data$no.fn),function(j) {
          if("glmmPQL" %in% class(globalmods.sub.list[[i]])) {
            mod=globalmods.sub.list[[i]]
            data.frame(
              threshold=i/100,
              Sys1=k,
              FTG=l,
              no.fn=j,
              Intercept=
                summary(mod)$tTable["(Intercept)","Value"]+
                ifelse(k=="Aquatic",0,summary(mod)$tTable[paste("Sys1",k,sep=""),"Value"])+
                ifelse(l=="Primary Producer",0,summary(mod)$tTable[paste("FTG",l,sep=""),"Value"])+
                j*summary(mod)$tTable["no.fn","Value"],
              Estimate=
                summary(mod)$tTable["richness","Value"]+
                ifelse(k=="Aquatic",0,summary(mod)$tTable[paste("richness:Sys1",k,sep=""),"Value"])+
                ifelse(l=="Primary Producer",0,summary(mod)$tTable[paste("richness:FTG",l,sep=""),"Value"])+
                j*summary(mod)$tTable["richness:no.fn","Value"] )
          } else {
            data.frame() } }
        ) }
    } ) } ) } )

#Plot coefs against threshold (subset out max number of functions): 9" x 5"
ggplot(
  data=ddply(subset(globalcoefs.sub.df,FTG %in% c("Primary Producer","Herbivore")),c("Sys1","FTG"),function(x)
subset(x,no.fn==max(no.fn))),
  aes(x=threshold,y=Estimate,col=Sys1,group=Sys1))+
  geom_rect(data=data.frame(
    FTG=levels(globalcoefs.sub.df$FTG)[c(1,5)],
    Sys1=rep(levels(globalcoefs.sub.df$Sys1),each=4),
    threshold=0,Estimate=0),
    aes(fill=FTG),xmin=-Inf,xmax=Inf,ymin=-Inf,ymax=Inf,alpha=0.15,show_guide=F)+
  scale_fill_manual(values=c("deepskyblue3","forestgreen"),guide="none")+
  geom_hline(xintercept=0,lwd=0.8,lty=1,col="grey30")+
  geom_line(lwd=1)+#aes(lty=Sys1),lwd=1)+
  #scale_linetype_manual(values=c(1,6))+
  scale_color_manual(values=c("blue2","darkgreen"),guide=guide_legend(ncol=1),name="")+
  facet_grid(~FTG,scales="free",space="free")+
  scale_x_continuous(breaks=c(0,0.5,1),labels=c("0","0.5","1"))+
  geom_text(data=data.frame(
    FTG=levels(globalcoefs.sub.df$FTG)[c(1,5)],
    Sys1=rep(levels(globalcoefs.sub.df$Sys1),each=4),
```

```
    labels=letters[1:2]),
    aes(x=0.1,y=Inf,label=labels),vjust=1.5,col="black",fontface="bold",size=6)+
  geom_text(data=data.frame(
    FTG=levels(globalcoefs.sub.df$FTG)[c(1,5)],
    Sys1=rep(levels(globalcoefs.sub.df$Sys1),each=4)),
    aes(x=0.15,y=-0.2,label=FTG),vjust=0,hjust=0,col="black",size=5)+
  labs(x="Threshold",y="Diversity Effect")+
  theme_bw(base_size=18)+
  theme(panel.grid.major=element_blank(),panel.grid.minor=element_blank(),
        strip.text.x=element_blank(),strip.background=element_blank(),
        legend.background=element_blank(),legend.key=element_blank(),
        legend.direction="horizontal",legend.box="horizontal",legend.position=c(0.12,0.77))

#####################################################################################################

#Simulations to explore the dip in diversity effect around 50% threshold for high numbers of functions
#Extract data for studies that exhibit the dip for further simulations
cedarcreek.df=subset(multifunc.long,Reference=="Cedar Creek (E120)" & value!="NA")
wardle.df=subset(multifunc.long,Reference=="Wardle et al. 2003" & value!="NA")

sim.df=ldply(1:100,.progress="text",function(rep) {
  ldply(seq(0,1,0.1),function(i) { #Percent of negative functions
    ldply(list(cedarcreek.df,wardle.df),function(j) {
      #Specify number of functions
      no.fn=length(unique(j$Ydesc))
      #Specify diversity levels
      divlevels=j[j$Ydesc %in% unique(j$Ydesc)[1],"richness"]
      #Construct data.frame with appropriate dims
      newdf=cbind(
        data.frame(diversity=divlevels),
        matrix(rep(NA,length(divlevels)*no.fn),nrow=length(divlevels)) )
      #Populate with values
      newdf[,2:ncol(newdf)]=colwise(function(x) rnorm(nrow(newdf),newdf$diversity,1))(newdf[,2:ncol(newdf)])
      #Define % of functions that have a negative relationship with diversity
      negcol=round(no.fn*i)
      #Convert that number in newdf to negative
      if(negcol>0) newdf[,2:(negcol+1)]=-newdf[,2:(negcol+1)]+max(newdf[,2:(negcol+1)]) else newdf=newdf
      #Scale response
      #newdf[,2:ncol(newdf)]=colwise(function(x) x/max(x))(newdf[,2:ncol(newdf)])
      #Generate threshold data
      thresh.df=ldply(1:99/100,function(k)
        cbind(
          diversity=newdf[,1],
          thresh=k,
          no.fn.greater=rowSums(colwise(function(x) x>=max(x)*k)(newdf[,2:ncol(newdf)])) ) )
      #Get linear slopes
      ddply(thresh.df,"thresh",function(x) {
        mod=try(glm(no.fn.greater~diversity,data=x,family=quasipoisson(link="identity"),start=c(0,0.1)))
        if(class(mod) == "try-error")
          cbind(
            rep=rep,
            Reference=as.character(unique(j$Reference)),
            pneg=i,
            no.fn=no.fn,
            thresh=unique(x$thresh),
            Estimate=NA,
            Std.Error=NA,
            N=NA) else
              cbind(
                rep=rep,
                Reference=as.character(unique(j$Reference)),
                pneg=i,
                no.fn=no.fn,
                thresh=unique(x$thresh),
                Estimate=summary(mod)$coefficients["diversity",1],
                Std.Error=summary(mod)$coefficients["diversity",2],
                n=mod$df.residuals) } )
    } )
  } )
} )

sim.df[,3:7]=apply(sim.df[,3:7],2,function(x) as.numeric(x))

#Summarize for plotting
sim.df.summary=ddply(sim.df,c("Reference","pneg","no.fn","thresh"),function(x)
  data.frame(
    Reference=unique(x$Reference),
    pneg=unique(x$pneg),
    no.fn=unique(x$no.fn),
    thresh=unique(x$thresh),
    Estimate=mean(x$Estimate),
    Std.Error=std.error(x$Estimate)) )
#    Std.Error=sqrt(sum(x$Std.Error/x$n)) ) )

#Graph results
ggplot(sim.df,aes(x=thresh,y=Estimate,group=rep))+
  geom_hline(yintercept=0,col="grey50",lwd=0.5)+
  #  geom_ribbon(aes(ymin=Estimate-Std.Error,ymax=Estimate+Std.Error),col="blue")+
```

```
  geom_line(lwd=0.3,alpha=0.7)+
  scale_x_continuous(breaks=c(0,0.5,1),labels=c("0","0.5","1"))+
  #scale_color_gradient(high="green",low="firebrick1",name="Number of\nfunctions")+
  facet_grid(Reference~pneg,scales="free")+
  labs(x="Threshold",y="Diversity effect")+
  theme_bw(base_size=18)+
  theme(panel.grid.major=element_blank(),panel.grid.minor=element_blank())

#####################################################################################################

#Conduct sensitivity analysis by removing each study individually, and re-generating coef plot
rawmods.sensitivity.df=ldply(c("0.2","0.4","0.6","0.8"),function(i) {
  ldply(unique(thresholds.df$Study),.progress="text",function(j) {
    #Subset data
    data=subset(thresholds.df,Study!=j & threshold==i)
    #Function to run models
    f=function(x) glmmPQL(no.fn.greater~richness*no.fn,random=~richness|Study,
                          family=quasipoisson(link="identity"),start=c(0.15,0.05,0.1,0.001),
                          control=lmeControl(opt="optim",msTol=1e-4),
                          verbose=F,data=x)
    #Run model, return NA if model fails
    safef=failwith(NA,f)
    mod=safef(data)
    #Extract coefficient and standard error and return in data.frame
    if(is.na(mod)) data.frame(Study.removed=j,threshold=i,coef=NA,coef.se=NA) else
      data.frame(
        Study.removed=j,
        threshold=i,
        coef=summary(mod)$tTable[2,1],
        coef.se=summary(mod)$tTable[2,2] )
  } )
} )

rawmods.sensitivity.df$Ref.removed=thresholds.df[match(rawmods.sensitivity.df$Study,thresholds.df$Study),"Refe
rence"]
rawmods.sensitivity.df$threshold=factor(rawmods.sensitivity.df$threshold)
levels(rawmods.sensitivity.df$threshold)=c("20%","40%","60%","80%")

#Plot results: 12" x 8"
ggplot(rawmods.sensitivity.df,aes(y=coef,x=Ref.removed,col=Ref.removed))+
  geom_hline(data=data.frame(
    threshold=c("20%","40%","60%","80%"),
    coef=c(summary(rawmods.list[[20]])$tTable[2,1],
           summary(rawmods.list[[40]])$tTable[2,1],
           summary(rawmods.list[[60]])$tTable[2,1],
           summary(rawmods.list[[80]])$tTable[2,1]) ),
    aes(yintercept=coef),lwd=1,alpha=0.6,lty=1)+
  geom_point(size=3)+
  geom_errorbar(aes(ymax=coef+2*coef.se,ymin=coef-2*coef.se))+
  coord_flip()+
  facet_wrap(~threshold,nrow=1)+
  labs(y="Regression coefficient",x="Study removed")+
  theme_bw(base_size=12)+
  theme(legend.position="none",panel.grid.major=element_blank(),panel.grid.minor=element_blank())

#####################################################################################################
#                                           TURNOVER APPROACH                                       #
#####################################################################################################

#First, extract only mono data for each experiment and store in a list
mono.list=dlply(multifunc,c("Reference","Study","Expt","Smax","Sys1","FTG"),function(i) {
  #Get monocultures and metadata
  monos=i[,c(2:4,9,grep("mono",colnames(i)))]
  #Melt monos dataframe
  monos.melt=melt(monos,id.vars=c(1:4),measures.vars=c(5:ncol(monos)))
  #Split columns based on response (Y, N, SD, or ID)
  monos.melt=cbind(monos.melt[1:4],
                   t(matrix(unlist(strsplit(gsub("([0-9]+)","\\1~",monos.melt$variable),"~" )),nrow=2)),
                   value=monos.melt[,"value"])
  names(monos.melt)[5:6]=c("treatment","variable")
  #Cast variables
  monos.cast=dcast(monos.melt,Study+Expt+Reference+Ydesc+treatment~variable,value.var="value")
  #Remove rows where Y==NA
  monos.cast=monos.cast[!is.na(monos.cast$Y),]
  #Convert responses to numeric
  monos.cast[,c("Y","SD","N")]=apply(monos.cast[,c("Y","SD","N")],2,as.numeric)
  #Return dataframe
  return(monos.cast)
} )

#Determine the most extreme species for each function, then sum the number of unique most extreme species
#across all functions within an experiment
extremesp.df=ldply(mono.list,function(i) {
  data.frame(no.sp=length(unique(i$treatment)),no.fn=length(unique(i$Ydesc)),
             no.max.sp=length(unique(ddply(i,"Ydesc",function(x) x[which.max(x$Y),"ID"])$V1)),
             no.min.sp=length(unique(ddply(i,"Ydesc",function(x) x[which.min(x$Y),"ID"])$V1)) ) } )

#Mean turnover in extreme species across all functions measured within an experiment
```

```
mean(extremesp.df$no.max.sp/extremesp.df$no.fn); std.error(extremesp.df$no.max.sp/extremesp.df$no.fn);
range(extremesp.df$no.max.sp/extremesp.df$no.fn)
mean(extremesp.df$no.min.sp/extremesp.df$no.fn); std.error(extremesp.df$no.min.sp/extremesp.df$no.fn);
range(extremesp.df$no.min.sp/extremesp.df$no.fn)

#Parse by system and trophic level
ddply(extremesp.df,"Sys1",function(x) data.frame(
  max.effect.size=mean(x$no.max.sp/x$no.fn),
  max.std.error=std.error(x$no.max.sp/x$no.fn),
  min.effect.size=mean(x$no.min.sp/x$no.fn),
  min.std.error=std.error(x$no.min.sp/x$no.fn) ) )

ddply(extremesp.df,"FTG",function(x) data.frame(
  max.effect.size=mean(x$no.max.sp/x$no.fn),
  max.std.error=std.error(x$no.max.sp/x$no.fn),
  min.effect.size=mean(x$no.min.sp/x$no.fn),
  min.std.error=std.error(x$no.min.sp/x$no.fn)  ) )

####################################################################################################
#                                       AVERAGING APPROACH                                         #
####################################################################################################

#Calculate average level of functioning across all functions for each treatment, for each experiment
multifunc.avg=ddply(multifunc.scaled,c("Reference","Study","Expt","FTG","Sys1","Sys2"),function(x) {
  z=data.frame(
    richness=colnames(x[,Y.colnames]),
    no.fn=length(unique(x$Ydesc)),
    avg.fn=colMeans(x[,Y.colnames]),
    avg.fn.SD=sqrt(
      colSums((x[,N.colnames]-1)*(x[,SD.colnames]^2),na.rm=T)/
        (colSums(x[,N.colnames],na.rm=T)-nrow(x[,N.colnames])) ),
    avg.fn.N=colSums(x[,N.colnames]) )
  #Remove rows where there is no response (i.e., avg.fn==NA)
  z=z[!is.na(z$avg.fn),]
  #Set richness by splitting column names
  z$richness=suppressWarnings(ifelse(grepl("mono",z$richness),1,as.numeric(gsub("X([0-
9]+).*","\\1",z$richness))))
  return(z) } )

#Investigate proper functional form to use
#Group data for random effects
multifunc.avg.grouped=groupedData(avg.fn~richness|Study,data=multifunc.avg)
#Fit different functional forms using non-linear mixed models
Null=nlme(avg.fn~a,fixed=a~1,random=~a~1,start=c(a=0.2),data=multifunc.avg.grouped)
Linear=nlme(avg.fn~a+b*richness,fixed=a+b~1,random=~a+b~1,start=c(a=1.5,b=1),data=multifunc.avg.grouped)
Logarithmic=nlme(avg.fn~a+b*log(richness),fixed=a+b~1,random=~a+b~1,start=c(a=1,b=1),data=multifunc.avg.groupe
d)
Power=nlme(avg.fn~a*richness^b,fixed=a+b~1,random=~a+b~1,start=c(a=0.2,b=2),data=multifunc.avg.grouped)
Saturating=nlme(avg.fn~richness/(k+richness),fixed=k~1,random=k~1,start=c(k=1),data=multifunc.avg.grouped)
#Compare models using AIC
AIC(Null,Linear,Logarithmic,Power,Saturating)

#Fit log relationship using linear mixed effects model, allowing slopes and intercepts to vary by Study
avgmods.list=lapply(c("unweighted","variance","sample.size"),function(i) {
  #Subset dataset to include only non-NA data points for each type of analysis
  if(i=="variance") { multifunc.avg=multifunc.avg[!is.na(multifunc.avg$avg.fn.SD),]
  } else if(i=="sample.size") { multifunc.avg=multifunc.avg[!is.na(multifunc.avg$avg.fn.N),]
  } else { multifunc.avg }
  #Fit linear mixed effects model for each weighting scheme
  if(i=="unweighted") {
    mod=glmmPQL(avg.fn~log(richness),random=~richness|Study,family=quasibinomial(link="identity"),
                start=c(0.5,0),data=multifunc.avg,verbose=F)
  } else if(i=="variance") {
    mod=glmmPQL(avg.fn~log(richness),random=~richness|Study,weights=1/((multifunc.avg$avg.fn.SD^2)+0.01),
                family=quasibinomial(link="identity"),start=c(0.5,0),data=multifunc.avg,verbose=F)
  } else {
    mod=glmmPQL(avg.fn~log(richness),random=~richness|Study,weights=sqrt(multifunc.avg$avg.fn.N),
                family=quasibinomial(link="identity"),start=c(0.5,0),data=multifunc.avg,verbose=F) }
  #Return model
  return(mod)
} )
#Append reduced model (S <= 16)
avgmods.list=append(avgmods.list,list(update(avgmods.list[[1]],data=subset(multifunc.avg,richness<=16))))
#Look at output and diagnostic plots
lapply(avgmods.list,summary); lapply(avgmods.list,plot)

#Extract predicted fits for plot
pred.df.list=lapply(seq_along(avgmods.list),function(i) {
  if(i<4) multifunc.avg=multifunc.avg else multifunc.avg=subset(multifunc.avg,!paste(Study,Expt) %in%

unique(paste(subset(multifunc.avg,richness>16)$Study,subset(multifunc.avg,richness>16)$Expt)))
  #Modified from: http://glmm.wikidot.com/faq
  #Create dataframe for predicted values for overall fit
  newdata=expand.grid(richness=1:max(multifunc.avg$richness),no.fn=2:max(multifunc.avg$no.fn),avg.fn=0)
  #Generate predicted values for overall trend
  newdata$avg.fn=predict(avgmods.list[[i]],newdata,type="response",level=0)
  #Obtain model matrix
  mm=model.matrix(terms(avgmods.list[[i]]),newdata)
```

```
  #Obtain estimate of SE based on fixed effects only
  newdata$fixedSE=sqrt(diag(mm %*% tcrossprod(vcov(avgmods.list[[i]]),mm)))

  #Create dataframe for predicted values for each study
  newdata2=ldply(unique(multifunc.avg$Study),function(j)
    data.frame(Study=j,
               richness=1:max(subset(multifunc.avg,Study==j)$richness),
               no.fn=max(subset(multifunc.avg,Study==j)$no.fn)) )
  #Generate predicted values for each study
  newdata2$avg.fn=predict(avgmods.list[[i]],newdata2,type="response",level=1)

  #Return dataframes in a list
  list(newdata,newdata2)
} )

#Plot predicted values and confidence bands across all studies on top of fitted values for each individual
study
avgplots.list=lapply(1:3,function(i) {
  ggplot()+
    #Plot raw points
    geom_point(data=multifunc.avg,aes(x=richness,y=avg.fn),size=2,col="grey60",alpha=0.5)+
    #Plot curves for each study
    geom_line(data=pred.df.list[[i]][[2]],aes(x=richness,y=avg.fn,group=Study),col="black",lwd=0.8,alpha=0.7)+
    #Add confidence band for mixed model predictions based on fixed effects only
    geom_ribbon(data=pred.df.list[[i]][[1]],
                aes(x=richness,y=avg.fn,ymax=avg.fn+2*fixedSE,ymin=avg.fn-2*fixedSE),
                fill="red",alpha=0.4)+
    #Add line for mixed model predictions
    geom_line(data=pred.df.list[[i]][[1]],aes(x=richness,y=avg.fn),col="red",lwd=1.5,alpha=0.9)+
    scale_x_continuous(breaks=c(1,20,40,60))+
    scale_y_continuous(limits=c(0,1.1),breaks=c(0,0.5,1))+
    labs(x="Richness",y="Average multifunctionality")+
    theme_bw(base_size=18)+
    theme(panel.grid.major=element_blank(),panel.grid.minor=element_blank())
} )#; avgplots.list

#Plot figure 1 with inset from list above (5 x 4.5")
p1=ggplot(data=subset(multifunc.avg,!paste(Study,Expt) %in%

unique(paste(subset(multifunc.avg,richness>16)$Study,subset(multifunc.avg,richness>16)$Expt))),
          aes(x=richness,y=avg.fn))+
  #Plot raw points
  geom_point(size=2.5,col="grey60",alpha=0.3)+
  #Plot curves for each study
  geom_line(data=pred.df.list[[4]][[2]],aes(x=richness,y=avg.fn,group=Study),col="grey20",lwd=0.75,alpha=0.8)+
  #Add confidence band for mixed model predictions based on fixed effects only
  geom_ribbon(data=pred.df.list[[4]][[1]],
              aes(x=richness,y=avg.fn,ymax=avg.fn+2*fixedSE,ymin=avg.fn-2*fixedSE),
              fill="red",alpha=0.4)+
  #Add line for mixed model predictions
  geom_line(data=pred.df.list[[4]][[1]],aes(x=richness,y=avg.fn),col="red",lwd=2.5)+
  coord_cartesian(ylim=c(-0.05,1.1))+
  scale_x_continuous(breaks=c(1,4,8,12,16),labels=c("1","4","8","12","16"))+
  scale_y_continuous(breaks=seq(0,1,0.2))+
  labs(x="Richness",y="Average multifunctionality")+
  geom_text(data=data.frame(
    labels=letters[1]),
    aes(x=-Inf,y=Inf,label=labels),vjust=1.5,hjust=-1.5,col="black",fontface="bold",size=9)+
  theme_bw(base_size=18)+
  theme(panel.grid.major=element_blank(),panel.grid.minor=element_blank())+

annotation_custom(grob=ggplotGrob(avgplots.list[[1]]+labs(x="",y="")+theme(plot.margin=unit(c(0,0,0,0),"cm")))
,
                    xmin=7,xmax=16,ymin=-0.075,ymax=0.385)

####################################################################################################

#Look at diversity effects on single functions
#Rearrange scaled responses so they are in the same format as the averaged dataset
multifunc.single=data.frame(
  melt(multifunc.scaled,
       id.vars=c("Reference","Study","Expt","FTG","Sys1","Sys2","Ydesc"),
       measure.vars=c(Y.colnames)),
  fn.N=melt(multifunc.scaled,
            id.vars=c("Reference","Study","Expt","FTG","Sys1","Sys2","Ydesc"),
            measure.vars=c(N.colnames))[9],
  fn.SD=melt(multifunc.scaled,
             id.vars=c("Reference","Study","Expt","FTG","Sys1","Sys2","Ydesc"),
             measure.vars=c(SD.colnames))[9] )
names(multifunc.single)[9:11]=c("fn.mean","fn.N","fn.SD")
multifunc.single=multifunc.single[!is.na(multifunc.single$fn.mean),]
multifunc.single$richness=
  suppressWarnings(ifelse(grepl("mono",multifunc.single$variable),1,as.numeric(gsub("X([0-
9]+).*","\\1",multifunc.single$variable))))

#Fit linear mixed effects model allowing slopes and intercepts to vary by Study
singlemods.list=lapply(c("unweighted","variance"),function(i) { #,"sample.size"),function(i) {
  #Subset dataset to include only non-NA data points for each type of analysis
```

```
  if(i=="variance") { multifunc.single=multifunc.single[!is.na(multifunc.single$fn.SD),]
  } else if(i=="sample.size") { multifunc.single=multifunc.single[!is.na(multifunc.single$fn.N),]
  } else { multifunc.single }
  #Fit linear mixed effects model for each weighting scheme
  if(i=="unweighted") {
    mod=glmmPQL(fn.mean~log(richness),random=~richness|Study/Ydesc,family=quasibinomial(link="identity"),
                start=c(0.5,0),control=lmeControl(msTol=1e-5,opt="optim"),data=multifunc.single,verbose=F)
  } else if(i=="variance") {

mod=glmmPQL(fn.mean~log(richness),random=~richness|Study/Ydesc,weights=1/((multifunc.single$fn.SD^2)+0.01),
                family=quasibinomial(link="identity"),start=c(0.5,0),control=lmeControl(msTol=1e-
5,opt="optim"),data=multifunc.single,verbose=F)
  } else {
    mod=glmmPQL(fn.mean~log(richness),random=~richness|Study/Ydesc,weights=sqrt(multifunc.single$fn.N),
                family=quasibinomial(link="identity"),start=c(0.2,0),control=lmeControl(msTol=1e-
5,opt="optim"),data=multifunc.single,verbose=F)
  }
  #Return model
  return(mod)
} )
#Look at output and diagnostic plots
lapply(singlemods.list,summary); lapply(singlemods.list,plot)

#Extract predicted fits for plot
singlepred.df.list=lapply(seq_along(singlemods.list),function(i) {
  #Modified from: http://glmm.wikidot.com/faq
  #Create dataframe for predicted values for overall fit
  newdata=expand.grid(richness=1:max(multifunc.single$richness),
                      fn.mean=0)
  #Generate predicted values for overall trend
  newdata$fn.mean=predict(singlemods.list[[i]],newdata,type="response",level=0)
  #Obtain model matrix
  mm=model.matrix(terms(singlemods.list[[i]]),newdata)
  #Obtain estimate of SE based on fixed effects only
  newdata$fixedSE=sqrt(diag(mm %*% tcrossprod(vcov(singlemods.list[[i]]),mm)))

  #Create dataframe for predicted values for each study
  newdata2=ldply(unique(multifunc.single$Study),function(j) {
    ldply(unique(subset(multifunc.single,Study==j)$Expt),function(k) {
      expand.grid(Study=j,Expt=k,
                  Ydesc=unique(subset(multifunc.single,Study==j)$Ydesc),
                  richness=1:max(subset(multifunc.single,Study==j)$richness)) } ) } )
  #Generate predicted values for each study
  newdata2$fn.mean=predict(singlemods.list[[i]],newdata2,type="response",level=2)
  newdata2$Ydesc=paste(newdata2$Ydesc,newdata2$Study,newdata2$Expt,sep=".")

  #Return dataframes in a list
  list(newdata,newdata2)
} )

#Plot predicted values and confidence bands across all studies on top of fitted values for each individual
study
singleplots.list=lapply(1:2,function(i) {
  ggplot()+
    #Plot raw points
    #geom_point(data=multifunc.single,aes(x=richness,y=fn.mean),size=2,col="grey60",alpha=0.5)+
    #Plot curves for each function
    #     geom_line(data=singlepred.df.list[[i]][[2]],aes(x=richness,y=fn.mean,group=Ydesc),
    #               alpha=0.3,lwd=0.8)+
    #Add confidence band for mixed model predictions based on fixed effects only
    geom_ribbon(data=singlepred.df.list[[i]][[1]],
                aes(x=richness,y=fn.mean,ymax=fn.mean+2*fixedSE,ymin=fn.mean-2*fixedSE),
                fill="red",alpha=0.3)+
    #Add line for mixed model predictions
    geom_line(data=singlepred.df.list[[i]][[1]],aes(x=richness,y=fn.mean),col="red",lwd=1.5,alpha=0.9)+
    scale_y_continuous(limits=c(0,1.1),breaks=seq(0,1,0.2))+
    labs(x="Richness",y="Functioning (single functions pooled)")+
    theme_bw(base_size=18)+
    theme(panel.grid.major=element_blank(),panel.grid.minor=element_blank())
} )

#Plot with average multifunctionality
p2=ggplot()+
  geom_point(data=multifunc.single,aes(x=richness,y=fn.mean),size=2,col="grey60",alpha=0.5)+
  #   geom_ribbon(data=pred.df.list[[1]][[1]],
  #               aes(x=richness,y=avg.fn,ymax=avg.fn+2*fixedSE,ymin=avg.fn-2*fixedSE),
  #               fill="red",alpha=0.4)+
  geom_ribbon(data=singlepred.df.list[[1]][[1]],
              aes(x=richness,y=fn.mean,ymax=fn.mean+2*fixedSE,ymin=fn.mean-2*fixedSE),
              fill="blue",alpha=0.3)+
  #Add line for mixed model predictions
  #   geom_line(data=pred.df.list[[1]][[1]],aes(x=richness,y=avg.fn),col="red",lwd=1.5,alpha=0.9)+
  geom_line(data=singlepred.df.list[[1]][[1]],aes(x=richness,y=fn.mean),col="blue",lwd=1.5,alpha=0.9)+
  #   scale_fill_manual(values=c("red","blue"),name="")+
  coord_cartesian(ylim=c(-0.05,1.1),xlim=c(-2,62))+
  scale_x_continuous(breaks=c(1,20,40,60))+
  scale_y_continuous(breaks=seq(0,1,0.2))+
  geom_text(data=data.frame(
```

```
    labels=letters[2]),
    aes(x=-Inf,y=Inf,label=labels),vjust=1.5,hjust=-1.5,col="black",fontface="bold",size=9)+
  labs(x="Richness",y="Scaled single functions")+
  theme_bw(base_size=18)+
  theme(panel.grid.major=element_blank(),panel.grid.minor=element_blank(),
        legend.position=c(0.9,0.3))

#13" x 6"
grid.arrange(p1,p2,nrow=1)

####################################################################################################

#Fit global mod using all predictors to see whether there are significant differences in the effect of
richness
#on multifunctionality across systems or trophic groups
global.mod=glmmPQL(avg.fn~log(richness)*no.fn+log(richness)*FTG+log(richness)*Sys1,random=~richness|Study,fami
ly=quasibinomial(link="identity"),
                   start=c(0.5,rep(0,13)),data=multifunc.avg,verbose=F)
summary(global.mod); plot(global.mod)

#Extract predicted fits for plot
predglobal.df=ldply(unique(multifunc.avg$Sys1),function(i) {
  ldply(unique(multifunc.avg$FTG),function(j) {
    if(nrow(subset(multifunc.avg,Sys1==i & FTG==j))==0) {
      data.frame()
    } else {
      #Generate dataframe for predicted values
      expand.grid(
        richness=1:max(subset(multifunc.avg,Sys1==i & FTG==j)$richness),
        no.fn=1:max(subset(multifunc.avg,Sys1==i & FTG==j)$no.fn),
        Sys1=i,
        FTG=j,
        avg.fn=NA)
    } } ) } )
#Generate predict values for overall trends
predglobal.df$avg.fn=predict(global.mod,predglobal.df,type="response",level=0)

#Plot the results
ggplot()+
  #Plot raw points
  geom_point(data=multifunc.avg,aes(x=richness,y=avg.fn),size=2,col="grey60",alpha=0.5)+
  #Add confidence band for mixed model predictions based on fixed effects only
  #   geom_ribbon(data=pred.df.list[[i]][[1]],
  #               aes(x=richness,y=avg.fn,fill=no.fn,group=no.fn,ymax=avg.fn+2*fixedSE,ymin=avg.fn-2*fixedSE),
  #               alpha=0.15)+
  #Add line for mixed model predictions
  geom_line(data=predglobal.df,aes(x=richness,y=avg.fn,col=no.fn,group=no.fn),lwd=1.5,alpha=0.8)+
  scale_color_gradient(high="red",low="blue",name="Number of\nfunctions")+
  #Add facets by Sys1 and FTG
  facet_grid(Sys1~FTG,scales="free")+#,ncol=2,nrow=5)+
  scale_y_continuous(breaks=seq(0,1,0.2))+
  labs(x="Richness",y="Average multifunctionality")+
  theme_bw(base_size=18)+
  theme(panel.grid.major=element_blank(),panel.grid.minor=element_blank(),strip.text.x=element_text(size=12),
        legend.position=c(0.075,0.25))

####################################################################################################

#Repeat, but include number of functions as an interaction
#Fit linear mixed effects model allowing slopes and intercepts to vary by Study
avgintmods.list=lapply(c("unweighted","variance","sample.size"),function(i) {
  #Subset dataset to include only non-NA data points for each type of analysis
  if(i=="variance") { multifunc.avg=multifunc.avg[!is.na(multifunc.avg$avg.fn.SD),]
  } else if(i=="sample.size") { multifunc.avg=multifunc.avg[!is.na(multifunc.avg$avg.fn.N),]
  } else { multifunc.avg }
  #Fit linear mixed effects model for each weighting scheme
  if(i=="unweighted") {
    mod=glmmPQL(avg.fn~log(richness)*no.fn,random=~richness|Study,family=quasibinomial(link="identity"),
                start=c(0.5,0,0,0),data=multifunc.avg,verbose=F)
  } else if(i=="variance") {

mod=glmmPQL(avg.fn~log(richness)*no.fn,random=~richness|Study,weights=1/((multifunc.avg$avg.fn.SD^2)+0.01),
                family=quasibinomial(link="identity"),start=c(0.5,0,0,0),data=multifunc.avg,verbose=F)
  } else {
    mod=glmmPQL(avg.fn~log(richness)*no.fn,random=~richness|Study,weights=sqrt(multifunc.avg$avg.fn.N),
                family=quasibinomial(link="identity"),start=c(0.5,0,0,0),data=multifunc.avg,verbose=F) }
  #Return model
  return(mod)
} )
#Look at output and diagnostic plots
lapply(avgintmods.list,summary); lapply(avgintmods.list,plot)

#Extract predicted fits for plot
predint.df.list=lapply(seq_along(avgintmods.list),function(i) {
  #Modified from: http://glmm.wikidot.com/faq
  #Create dataframe for predicted values for overall fit
  newdata=expand.grid(richness=1:max(multifunc.avg$richness),
                      no.fn=2:max(multifunc.avg$no.fn),
```

```
                       avg.fn=0)
  #Generate predicted values for overall trend
  newdata$avg.fn=predict(avgintmods.list[[i]],newdata,type="response",level=0)
  #Obtain model matrix
  mm=model.matrix(terms(avgintmods.list[[i]]),newdata)
  #Obtain estimate of SE based on fixed effects only
  newdata$fixedSE=sqrt(diag(mm %*% tcrossprod(vcov(avgintmods.list[[i]]),mm)))
  #Return dataframe
  return(newdata)
} )

#Plot predicted values and confidence bands across all studies on top of fitted values for each individual
study
lapply(1:3,function(i) {
  ggplot()+
    #Plot raw points
    geom_point(data=multifunc.avg,aes(x=richness,y=avg.fn),size=2,col="grey60",alpha=0.5)+
    #Plot curves for each study
    #geom_line(data=pred.df.list[[i]][[2]],aes(x=richness,y=avg.fn,group=Study),alpha=0.75,lwd=1,alpha=0.75)+
    #Add confidence band for mixed model predictions based on fixed effects only
    geom_ribbon(data=predint.df.list[[i]],
                aes(x=richness,y=avg.fn,ymax=avg.fn+2*fixedSE,ymin=avg.fn-2*fixedSE,group=no.fn,fill=no.fn),
                alpha=0.15)+
    #Add line for mixed model predictions
    geom_line(data=predint.df.list[[i]],aes(x=richness,y=avg.fn,group=no.fn,col=no.fn),lwd=1.5,alpha=0.9)+
    scale_color_gradient(high="red",low="blue",name="Number of\nfunctions")+
    scale_fill_gradient(high="red",low="blue",name="Number of\nfunctions")+
    scale_y_continuous(breaks=seq(0,1,0.2))+
    labs(x="\nRichness",y="Average multifunctionality\n")+
    theme_bw(base_size=18)+
    theme(panel.grid.major=element_blank(),panel.grid.minor=element_blank())
} )

#Extract effect sizes and their standard errors for each level of number of functions
avginteffect.df.list=lapply(avgintmods.list,function(i) {
  ldply(2:max(multifunc.avg$no.fn),function(j)
    data.frame(
      no.fn=j,
      Estimate=summary(i)$tTable["log(richness)","Value"]+j*summary(i)$tTable["log(richness):no.fn","Value"],
      Std.Error=sqrt(
(summary(i)$tTable["log(richness)","Std.Error"]^2+summary(i)$tTable["log(richness):no.fn","Std.Error"]^2+
                         2*i$varFix["log(richness)","log(richness):no.fn"]) ) )
  ) } )

#Plot number of functions against effect size
lapply(avginteffect.df.list,function(i) {
  ggplot(i,aes(x=no.fn,y=Estimate))+
    #Add points for effect sizes
    geom_point(size=4)+
    #And error bars for the standard errors
    geom_errorbar(aes(ymax=Estimate+2*Std.Error,ymin=Estimate-2*Std.Error),width=0)+
    #Specify axis breaks
    scale_x_continuous(breaks=c(2,4,6,8,10,12))+
    labs(x="\nNumber of functions",y="Diversity effect (+/- 95% CI)\n")+
    theme_bw(base_size=18)+
    theme(panel.grid.major=element_blank(),panel.grid.minor=element_blank())
} )

##################################################################################################
#                                     MULTIPLICATIVE APPROACH                                    #
##################################################################################################

#Calculate multiplicative level of functioning across all functions for each treatment, for each experiment
multifunc.mult=ddply(multifunc.scaled,c("Reference","Study","Expt","FTG","Sys1","Sys2"),function(x) {
  z=data.frame(
    richness=colnames(x[,Y.colnames]),
    no.fn=length(unique(x$Ydesc)),
    mult.fn=apply(x[,Y.colnames],2,prod),
    mult.fn.N=colSums(x[,N.colnames]) )
  #Remove rows where there is no response (i.e., mult.fn.scaled==NA)
  z=z[!is.na(z$mult.fn),]
  #Scale by nth root
  z$mult.fn.scaled=z$mult.fn^(1/nrow(x))
  #Set richness by splitting column names
  z$richness=suppressWarnings(ifelse(grepl("mono",z$richness),1,as.numeric(gsub("X([0-
9]+).*","\\1",z$richness))))
  return(z) } )

#Investigate proper functional form to use
#Group data for random effects
multifunc.mult.grouped=groupedData(mult.fn.scaled~richness|Study,data=multifunc.mult)
#Fit different functional forms using non-linear mixed models
Null=nlme(mult.fn.scaled~a,fixed=a~1,random=~a~1,start=c(a=0.2),data=multifunc.mult.grouped)
Linear=nlme(mult.fn.scaled~a+b*richness,fixed=a+b~1,random=~a+b~1,start=c(a=1.5,b=1),data=multifunc.mult.group
ed)
Logarithmic=nlme(mult.fn.scaled~a+b*log(richness),fixed=a+b~1,random=~a+b~1,start=c(a=1.5,b=1),data=multifunc.
mult.grouped)
```

```
Power=nlme(mult.fn.scaled~a*richness^b,fixed=a+b~1,random=~a+b~1,start=c(a=0.2,b=2),data=multifunc.mult.groupe
d)
Saturating=nlme(mult.fn.scaled~richness/(k+richness),fixed=k~1,random=k~1,start=c(k=1),data=multifunc.mult.gro
uped)
#Compare models using AIC
AIC(Null,Linear,Logarithmic,Power,Saturating)

#Fit log relationship using linear mixed effects model, allowing slopes and intercepts to vary by Study
multmods.list=lapply(c("unweighted"),function(i) {
  #Subset dataset to include only non-NA data points for each type of analysis
  if(i=="variance") { multifunc.mult=multifunc.mult[!is.na(multifunc.mult$mult.fn.scaled.SD),]
  } else if(i=="sample.size") { multifunc.mult=multifunc.mult[!is.na(multifunc.mult$mult.fn.scaled.N),]
  } else { multifunc.mult }
  #Fit linear mixed effects model for each weighting scheme
  if(i=="unweighted") {
    mod=glmmPQL(mult.fn.scaled~log(richness),random=~richness|Study,family=quasibinomial(link="identity"),
                start=c(0.5,0),data=multifunc.mult,verbose=F)
  } else if(i=="variance") {

mod=glmmPQL(mult.fn.scaled~log(richness),random=~richness|Study,weights=1/((multifunc.mult$mult.fn.scaled.SD^2
)+0.01),
                family=quasibinomial(link="identity"),start=c(0.5,0),data=multifunc.mult,verbose=F)
  } else {

mod=glmmPQL(mult.fn.scaled~log(richness),random=~richness|Study,weights=sqrt(multifunc.mult$mult.fn.scaled.N),
                family=quasibinomial(link="identity"),start=c(0.5,0),data=multifunc.mult,verbose=F) }
  #Return model
  return(mod)
} )
#Append reduced model (S <= 16)
multmods.list=append(multmods.list,list(update(multmods.list[[1]],data=subset(multifunc.mult,richness<=16))))
#Look at output and diagnostic plots
lapply(multmods.list,summary); lapply(multmods.list,plot)

#Extract predicted fits for plot
pred.df.list=lapply(seq_along(multmods.list),function(i) {
  if(i==1) multifunc.mult=multifunc.mult else multifunc.mult=subset(multifunc.mult,!paste(Study,Expt) %in%

unique(paste(subset(multifunc.mult,richness>16)$Study,subset(multifunc.mult,richness>16)$Expt)))
  #Modified from: http://glmm.wikidot.com/faq
  #Create dataframe for predicted values for overall fit

newdata=expand.grid(richness=1:max(multifunc.mult$richness),no.fn=2:max(multifunc.mult$no.fn),mult.fn.scaled=0
)
  #Generate predicted values for overall trend
  newdata$mult.fn.scaled=predict(multmods.list[[i]],newdata,type="response",level=0)
  #Obtain model matrix
  mm=model.matrix(terms(multmods.list[[i]]),newdata)
  #Obtain estimate of SE based on fixed effects only
  newdata$fixedSE=sqrt(diag(mm %*% tcrossprod(vcov(multmods.list[[i]]),mm)))

  #Create dataframe for predicted values for each study
  newdata2=ldply(unique(multifunc.mult$Study),function(j)
    data.frame(Study=j,
               richness=1:max(subset(multifunc.mult,Study==j)$richness),
               no.fn=max(subset(multifunc.mult,Study==j)$no.fn)) )
  #Generate predicted values for each study
  newdata2$mult.fn.scaled=predict(multmods.list[[i]],newdata2,type="response",level=1)

  #Return dataframes in a list
  list(newdata,newdata2)
} )

#Plot predicted values and confidence bands across all studies on top of fitted values for each individual
study
avgplots.list=lapply(1,function(i) {
  ggplot()+
    #Plot raw points
    geom_point(data=multifunc.mult,aes(x=richness,y=mult.fn.scaled),size=2,col="grey60",alpha=0.5)+
    #Plot curves for each study

geom_line(data=pred.df.list[[i]][[2]],aes(x=richness,y=mult.fn.scaled,group=Study),col="black",lwd=0.8,alpha=0
.7)+
    #Add confidence band for mixed model predictions based on fixed effects only
    geom_ribbon(data=pred.df.list[[i]][[1]],
                aes(x=richness,y=mult.fn.scaled,ymax=mult.fn.scaled+2*fixedSE,ymin=mult.fn.scaled-2*fixedSE),
                fill="red",alpha=0.4)+
    #Add line for mixed model predictions
    geom_line(data=pred.df.list[[i]][[1]],aes(x=richness,y=mult.fn.scaled),col="red",lwd=1.5,alpha=0.9)+
    scale_x_continuous(breaks=c(1,20,40,60))+
    scale_y_continuous(limits=c(0,1.1),breaks=c(0,0.5,1))+
    labs(x="Richness",y="Average multifunctionality")+
    theme_bw(base_size=18)+
    theme(panel.grid.major=element_blank(),panel.grid.minor=element_blank())
} )#; avgplots.list

#Plot figure 1 with inset from list above (5 x 4.5")
ggplot(data=subset(multifunc.mult,!paste(Study,Expt) %in%
```

```
unique(paste(subset(multifunc.mult,richness>16)$Study,subset(multifunc.mult,richness>16)$Expt))),
       aes(x=richness,y=mult.fn.scaled))+
  #Plot raw points
  geom_point(size=2.5,col="grey60",alpha=0.3)+
  #Plot curves for each study

geom_line(data=pred.df.list[[2]][[2]],aes(x=richness,y=mult.fn.scaled,group=Study),col="grey20",lwd=0.75,alpha
=0.8)+
  #Add confidence band for mixed model predictions based on fixed effects only
  geom_ribbon(data=pred.df.list[[2]][[1]],
              aes(x=richness,y=mult.fn.scaled,ymax=mult.fn.scaled+2*fixedSE,ymin=mult.fn.scaled-2*fixedSE),
              fill="red",alpha=0.4)+
  #Add line for mixed model predictions
  geom_line(data=pred.df.list[[2]][[1]],aes(x=richness,y=mult.fn.scaled),col="red",lwd=2.5)+
  coord_cartesian(ylim=c(-0.05,1.1))+
  scale_x_continuous(breaks=c(1,4,8,12,16),labels=c("1","4","8","12","16"))+
  scale_y_continuous(breaks=seq(0,1,0.2))+
  labs(x="Richness",y="Multiplicative multifunctionality")+
  #   geom_text(data=data.frame(
  #     labels=letters[1]),
  #     aes(x=-Inf,y=Inf,label=labels),vjust=1.5,hjust=-1.5,col="black",fontface="bold",size=9)+
  theme_bw(base_size=18)+
  theme(panel.grid.major=element_blank(),panel.grid.minor=element_blank())+

annotation_custom(grob=ggplotGrob(avgplots.list[[1]]+labs(x="",y="")+theme(plot.margin=unit(c(0,0,0,0),"cm")))
,
                    xmin=7,xmax=16,ymin=-0.075,ymax=0.385)
```

Supplementary References

1. Tilman, D., Reich, P. B. & Knops, J. M. H. Biodiversity and ecosystem stability in a decade-long grassland experiment. *Nature* **441,** 629–632 (2006).

2. Wardle, D. A., Yeates, G. W., Williamson, W. & Bonner, K. I. The response of a three trophic level soil food web to the identity and diversity of plant species and functional groups. *Oikos* **1,** 45–56 (2003).

3. Ball, B. A., Bradford, M. A., Coleman, D. C. & Hunter, M. D. Linkages between below and aboveground communities: Decomposer responses to simulated tree species loss are largely additive. *Soil Biol. Biochem.* **41,** 1155–1163 (2009).

4. Bardgett, R. D. & Shine, A. Linkages between plant litter diversity, soil microbial biomass and ecosystem function in temperate grasslands. *Soil Biol. Biochem.* **31,** 317–321 (1999).

5. Reich, P. B. *et al.* Impacts of biodiversity loss escalate through time as redundancy fades. *Science* **336,** 589–592 (2012).

6. Hector, A. *et al.* Plant diversity and productivity experiments in European grasslands. *Science* **286,** 1123–1127 (1999).

7. Blair, J. M., Parmelee, R. W. & Beare, M. H. Decay rates, nitrogen fluxes, and decomposer communiies of single-and mixed-species foliar litter. *Ecology* **71,** 1976–1985 (1990).

8. Boyer, K. E., Kertesz, J. S. & Bruno, J. F. Biodiversity effects on productivity and stability of marine macroalgal communities: the role of environmental context. *Oikos* **118,** 1062–1072 (2009).

9. Bruno, J. F. & O'Connor, M. I. Cascading effects of predator diversity and omnivory in a marine food web. *Ecol. Lett.* **8,** 1048–1056 (2005).

10. Caliman, A. *et al.* Functional bioturbator diversity enhances benthic-pelagic processes and properties in experimental microcosms. *J. North Am. Benthol. Soc.* **26,** 450–459 (2007).

11. Cardinale, B. J. & Palmer, M. A. Disturbance Moderates Biodiversity–Ecosystem Function Relationships: Experimental Evidence From Caddisflies in Stream Mesocosms. *Ecology* **83,** 1915–1927 (2002).

12. Dang, C. K., Chauvet, E. & Gessner, M. O. Magnitude and variability of process rates in fungal diversity-litter decomposition relationships. *Ecol. Lett.* **8,** 1129–1137 (2005).

13. De Deyn, G. B. *et al.* Vegetation composition promotes carbon and nitrogen storage in model grassland communities of contrasting soil fertility. *J. Ecol.* **97,** 864–875 (2009).

14. Douglass, J. G., Duffy, J. E. & Bruno, J. F. Herbivore and predator diversity interactively affect ecosystem properties in an experimental marine community. *Ecol. Lett.* **11,** 598–608 (2008).

15. Duarte, S., Pascoal, C., Cássio, F. & Bärlocher, F. Aquatic hyphomycete diversity and identity affect leaf litter decomposition in microcosms. *Oecologia* **147,** 658–666 (2006).

16. Duffy, J. E., Richardson, J. P. & Canuel, E. A. Grazer diversity effects on ecosystem functioning in seagrass beds. *Ecol. Lett.* **6,** 637–645 (2003).

17. Duffy, J. E., Paul Richardson, J. & France, K. E. Ecosystem consequences of diversity depend on food chain length in estuarine vegetation. *Ecol. Lett.* **8,** 301–309 (2005).

18. Dzialowski, A. R. & Smith, V. H. Nutrient dependent effects of consumer identity and diversity on freshwater ecosystem function. *Freshw. Biol.* **53,** 148–158 (2007).

19. Gamfeldt, L., Hillebrand, H. & Jonsson, P. R. Species richness changes across two trophic levels simultaneously affect prey and consumer biomass. *Ecol. Lett.* **8,** 696–703 (2005).

20. Godbold, J. A., Solan, M. & Killham, K. Consumer and resource diversity effects on marine macroalgal decomposition. *Oikos* **118,** 77–86 (2009).

21. Hägele, B. F. & Rowell-Rahier, M. Dietary mixing in three generalist herbivores: nutrient complementation or toxin dilution? *Oecologia* **119,** 521–533 (1999).

22. Hättenschwiler, S. & Bretscher, D. Isopod effects on decomposition of litter produced under elevated CO2, N deposition and different soil types. *Glob. Chang. Biol.* **7,** 565–579 (2001).

23. Hillebrand, H., Gamfeldt, L., Jonsson, P. R. & Matthiessen, B. Consumer diversity indirectly changes prey nutrient content. *Mar. Ecol. Prog. Ser.* **380,** 33–41 (2009).

24. Allan, E. *et al.* A comparison of the strength of biodiversity effects across multiple functions. *Oecologia* **173,** 223–237 (2013).

25. Jiang, L. Negative selection effects suppress relationships between bacterial diversity and ecosystem functioning. *Ecology* **88,** 1075–1085 (2007).

26. Karanika, E. D., Alifragis, D. A., Mamolos, A. P. & Veresoglou, D. S. Differentiation between responses of primary productivity and phosphorus exploitation to species richness. *Plant Soil* **297,** 69–81 (2007).

27. Keith, A. M. *et al.* Increasing litter species richness reduces variability in a terrestrial decomposer system. *Ecology* **89,** 2657–2664 (2008).

28. King, R. F., Dromph, K. M. & Bardgett, R. D. Changes in species evenness of litter have no effect on decomposition processes. *Soil Biol. Biochem.* **34,** 1959–1963 (2002).

29. Laossi, K.-R. *et al.* Effects of plant diversity on plant biomass production and soil macrofauna in Amazonian pastures. *Pedobiologia.* **51,** 397–407 (2008).

30. Madritch, M. D. & Cardinale, B. J. Impacts of tree species diversity on litter decomposition in northern temperate forests of Wisconsin, USA: a multi-site experiment along a latitudinal gradient. *Plant Soil* **292,** 147–159 (2007).

31. Maestre, F. T. & Reynolds, J. F. Biomass responses to elevated CO2, soil heterogeneity and diversity: an experimental assessment with grassland assemblages. *Oecologia* **151,** 512–520 (2007).

32. McKie, B. G. *et al.* Ecosystem functioning in stream assemblages from different regions: contrasting responses to variation in detritivore richness, evenness and density. *J. Anim. Ecol.* **77,** 495–504 (2008).

33. McKie, B. G., Schindler, M., Gessner, M. O. & Malmqvist, B. Placing biodiversity and ecosystem functioning in context: environmental perturbations and the effects of species richness in a stream field experiment. *Oecologia* **160,** 757–70 (2009).

34. Meier, C. L. & Bowman, W. D. Links between plant litter chemistry, species diversity, and below-ground ecosystem function. *PNAS* **105,** 19780–19785 (2008).

35. Nilsson, E. *et al.* Effects of stream predator richness on the prey community and ecosystem attributes. *Oecologia* **157,** 641–651 (2008).

36. Power, L. D. & Cardinale, B. J. Species richness enhances both algal biomass and rates of oxygen production in aquatic microcosms. *Oikos* **118,** 1703–1711 (2009).

37. Redondo-Brenes, A. & Montagnini, F. Growth, productivity, aboveground biomass, and carbon sequestration of pure and mixed native tree plantations in the Caribbean lowlands of Costa Rica. *For. Ecol. Manage.* **232,** 168–178 (2006).

38. Ritchie, M. E. & Tilman, D. Predictions of species interactions from consumer-resource theory: experimental tests with grasshoppers and plants. *Oecologia* **94,** 516–527 (1993).

39. Rixen, C. & Mulder, C. P. H. Improved water retention links high species richness with increased productivity in arctic tundra moss communities. *Oecologia* **146,** 287–299 (2005).

40. Sanpera-Calbet, I., Lecerf, A. & Chauvet, E. Leaf diversity influences in-stream litter decomposition through effects on shredders. *Freshw. Biol.* **54,** 1671–1682 (2009).

41. Spaekova, I. & Leps, J. Procedure for separating the selection effect from other effects in diversity-productivity relationship. *Ecol. Lett.* **4,** 585–594 (2001).

42. Stachowicz, J. J., Graham, M., Bracken, M. E. S. & Szoboszlai, A. I. Diversity enhances cover and stability of seaweed assemblages: the role of heterogeneity and time. *Ecology* **89,** 3008–3019 (2008).

43. Sullivan, G. & Zedler, J. B. Functional redundancy among tidal marsh halophytes: a test. *Oikos* **84,** 246–260 (1999).

44. Sullivan, G., Callaway, J. C. & Zedler, J. B. Plant assemblage composition explains and predicts how biodiversity affects salt marsh functioning. *Ecol. Monogr.* **77,** 569–590 (2007).

45. Van Peer, L., Nijs, I., Reheul, D. & De Cauwer, B. Species richness and susceptibility to heat and drought extremes in synthesized grassland ecosystems: compositional vs physiological effects. *Funct. Ecol.* **18,** 769–778 (2004).

46. Von Felten, S. & Schmid, B. Complementarity among species in horizontal versus vertical rooting space. *J. Plant Ecol.* **1,** 33–41 (2008).

47. Wojdak, J. M. Relative strength of top-down, bottom-up, and consumer species richness effects on pond ecosystems. *Ecol. Monogr.* **75,** 489–504 (2005).
